# Emerging trends in the development of flexible optrode arrays for electrophysiology

**DOI:** 10.1063/5.0153753

**Published:** 2023-09-07

**Authors:** Reem M. Almasri, François Ladouceur, Damia Mawad, Dorna Esrafilzadeh, Josiah Firth, Torsten Lehmann, Laura A. Poole-Warren, Nigel H. Lovell, Amr Al Abed

**Affiliations:** 1 Graduate School of Biomedical Engineering, UNSW, Sydney, NSW 2052, Australia; 2 School of Electrical Engineering and Telecommunications, UNSW, Sydney, NSW 2052, Australia; 3 Australian National Fabrication Facility, UNSW, Sydney, NSW 2052, Australia; 4 School of Materials Science and Engineering, UNSW, Sydney, NSW 2052, Australia; 5 Tyree Institute of Heath Engineering, UNSW, Sydney, NSW 2052, Australia

## Abstract

Optical-electrode (optrode) arrays use light to modulate excitable biological tissues and/or transduce bioelectrical signals into the optical domain. Light offers several advantages over electrical wiring, including the ability to encode multiple data channels within a single beam. This approach is at the forefront of innovation aimed at increasing spatial resolution and channel count in multichannel electrophysiology systems. This review presents an overview of devices and material systems that utilize light for electrophysiology recording and stimulation. The work focuses on the current and emerging methods and their applications, and provides a detailed discussion of the design and fabrication of flexible arrayed devices. Optrode arrays feature components non-existent in conventional multi-electrode arrays, such as waveguides, optical circuitry, light-emitting diodes, and optoelectronic and light-sensitive functional materials, packaged in planar, penetrating, or endoscopic forms. Often these are combined with dielectric and conductive structures and, less frequently, with multi-functional sensors. While creating flexible optrode arrays is feasible and necessary to minimize tissue–device mechanical mismatch, key factors must be considered for regulatory approval and clinical use. These include the biocompatibility of optical and photonic components. Additionally, material selection should match the operating wavelength of the specific electrophysiology application, minimizing light scattering and optical losses under physiologically induced stresses and strains. Flexible and soft variants of traditionally rigid photonic circuitry for passive optical multiplexing should be developed to advance the field. We evaluate fabrication techniques against these requirements. We foresee a future whereby established telecommunications techniques are engineered into flexible optrode arrays to enable unprecedented large-scale high-resolution electrophysiology systems.

## I. INTRODUCTION

Modulating and deciphering the electrophysiological functions and behaviors of electrically excitable cells can be acheived through a variety of techniques aimed at probing and bridging the signaling complexity of the biological networks. Most of these approaches employ stimulating/recording electrodes and conductive tracks, which are normally designed in the form of arrays denoted as microelectrode arrays (MEAs), with or without embedded micro-electronic circuitry. While these electrical interfaces can be designed in mechanically flexible shapes and configurations to enable direct contact with soft tissues, electrical recording/stimulation imposes fundamental challenges that limit long-term clinical and research applications. For instance, there is a fundamental trade-off between the size of contact size and functionality. A reduction in electrode size will (1) increase the contact interface impedance, necessitating amplifiers with high input impedances relative to the contact impedance to preserve recorded signal quality, and (2) reduce charge injection limits for stimulation. These limitations significantly restrict spatial resolution and contribute to undesirable chemical reactions at the interface, which in implants can lead to tissue damage, pH changes, and toxic by-products. Another fundamental constraint on electrical MEAs is wiring; a single conductive track can only transmit an analog signal sensed from a single contact site/electrode, thereby imposing constraints on the packaging and routing of wires to external data acquisition systems as the number of channels increases. This one-to-one ratio can be only overcome by introducing multiplexing circuitry or digitalizing the analog signals. The challenges for electrical recording revolve around the low spatial resolution and the susceptibility of the recorded signals to contamination by various noise sources and artifacts. These factors have contributed to the widening gap between MEA technologies being developed in research laboratories around the world and featuring tens of thousands of channels for acute preclinical studies, vs chronically implanted devices, and regulatory-approved and on-market clinical devices, which are limited to tens of channels.

Optical technologies have emerged as a promising alternative to tackle these challenges, offering new avenues for tissue stimulation and recording. A single beam of light can carry information from multiple channels by encoding signals in different channels using color/wavelength. Delivering and receiving light through the target tissues could, in principle, improve spatial resolution and selectivity, by precisely targeting specific cells or regions, and reduce background noise and artifacts like electromagnetic interference. In the past few years, great strides have been made in the development of these optical techniques, including but not limited to, calcium- and voltage-sensitive imaging using fluorophore dyes or genetic indicators, optogenetics, multifunctional systems with optical recording or stimulation capabilities, biopotential electro-optical transducers based on liquid crystals, phase-sensitive plasmonics, organic and inorganic photostimulation, and more. Most of these systems could be designed as arrays of multiple channels to achieve multisite activation or detection, which could typically combine optical fibers, waveguides, micro-LEDs (*μ*LEDs), light-activated and -modulated functional materials, with or without electrical modalities.

For clinical translation, any emerging or putative interfacial optical device needs to be encapsulated into implantable forms such that it will function chronically throughout the implant's life while minimizing adverse tissue response. In the case of brain implants, mechanical stability and conformability are important aspects to avoid disruption in the blood–brain barrier and damage to the blood vessels and surrounding tissues even in acute studies.^1^ Therefore, these optical technologies have an underlying need for mechanically robust and soft tissue-interfacing, which in turn has driven growing interest in designing flexible components. However, most of the relevant research studies have focused on electrical interfaces only.^1^ While many design considerations for flexible optical interfaces are often interrelated with that of the electrical interfaces, it is important to have a comprehensive understanding of how optical requirements play a role in the design and structure of the device and how fabrication techniques can vary accordingly.^2^

This review is intended for anyone interested in flexible light-based multichannel devices for electrophysiology applications; whether it be biomedical engineers, material scientists, photonic physicists, or biologists seeking light-based methodologies to further their understanding of the body's electrophysiology systems.

We surveyed the literature for original research papers, including peer-reviewed conference proceedings, as well as reviews published over the last ten years. In addition, we included earlier foundation and fundamental studies establishing various techniques. PubMed and Google Scholar were used as search engines as well as Clinicaltrials.gov and the Australian Register of Therapeutic Goods. Search terms included, but were not limited to, “optrode,” “optoelectronic,” “optical electrode,” “optical array,” “electro-optical array,” “upconversion nanoparticle,” “optogenetics array,” “infrared/NIR stimulation/modulation,” “quantum dots neural/cardiac/muscle,” “nanoparticle neural/cardiac/muscle stimulation/pacing,” “photovoltaic stimulation,” “photothermal stimulation,” “optical imaging/mapping endoscope electrophysiology,” “endoscopic/catheter optical array brain/heart/nerve,” “optical array catheter/basket/multimodal,” “multifunctional array light,” “multifunctional optical array,” “plasmonic arrays,” “multimodal arrays,” and “neuroplasmonics.”

Previous reviews have discussed the recent advances in implantable optogenetic interfaces along with some manufacturing and design challenges.^3–6^ Other review papers have focused on the biological considerations of optical interfaces for neuromodulation applications without consideration of their structural and mechanical properties.^7^ However, there are several technical challenges specifically associated with developing flexible optical modalities that must be met to enable reliable soft light-based array interfaces for electrophysiology. The present review will investigate the recent advances in fabrication techniques and materials to achieve flexible optical arrays for electrophysiology modulation and recording.

We narrowed our discussion to soft or flexible optical arrays; featuring multiple channels for recording and/or stimulation or material systems currently realized in single channel forms but for which we do not foresee any technical obstacles preventing array embodiments in the future.

Studies that were deemed outside of scope of this review include (1) optical transmission of data from one instrument to another as these do not interface directly with biological specimens, for example, optical links from preamplifiers to data acquisition systems, (2) conventional microscopy systems, and transparent multi-electrode arrays. Although this combination allows light transmission for the detection of fluorescent signals or excitation of fluorophores using light from a conventional microscope, the arrays do not act as waveguides and, therefore, are not inherently capable of targeted delivery of light; this functionality is provided by beam steering mechanisms and optics of the microscope system itself, (3) rigid optical arrays, as this Review focuses on fabrication challenges related to soft and flexible devices, and (4) conventional MEAs with no multichannel optical components; these devices are not light-based technologies.

To acquaint the reader with the basic terminology used in the field, we begin the review by defining the term optical-electrodes or optrodes, which refers to material systems, constructs, or devices that utilize light to

(1) sense or transduce electrophysiological signals from cells or tissues, or
(2) transmit such signals from the biological interface to data acquisition systems, or
(3) stimulate an electrical response in biological cells or tissues. The mechanism for eliciting such a response could be optical (e.g., retinal stimulation and optogenetics activation), photothermal, or optoelectronic (conversion of light into an electrical stimulus).

It is important to note that not all optical-based technologies in electrophysiology fall under the classification of optrodes. For instance, conventional microscopy systems and transparent multi-electrode arrays utilized to facilitate optical-based sensing or stimulation are not considered as optrodes within the context of this manuscript. Optrode arrays are multichannel systems in which the optrodes function as the sole elements or are integrated with other elements such as micro-electrodes, strain, temperature, and pH sensors.

This review will begin by providing a broad overview of the application of optrodes in electrophysiology, discussing both established and emerging sensing and stimulation technologies in Sec. III. Section IV describes the architectures and designs used for soft and flexible optrode arrays and devices. Section V identifies the common components of flexible optrode arrays by exploring the fabrication methods and classes of materials used for each component and mapping these to different applications and targeted tissues. Section VI examines the design considerations, key challenges, and technical requirements that must be addressed for clinical translation and commercial manufacturing of soft and flexible optrode arrays for acute and chronic applications. Finally, Sec. VII describes future directions covering the existing gaps, in terms of novel materials and fabrication options for emerging optical technologies.

## II. KEY APPLICATION AREAS

With our increased understanding of how local failure of bioelectrical signaling can give rise to many disease conditions, and sensory and motor impairments, the clinical diagnostic and therapeutic potential of multichannel electrophysiology systems is undeniable. Regulatory-approved instrumentation has contributed to reducing the translation barriers between preclinical research and human healthcare for many wire-based multi-channel recording or stimulating systems. Popular examples include invasive and noninvasive arrays for mapping the electrical activity of the brain and heart, deep brain stimulation for Parkinson's disease, spinal cord stimulation for chronic neuropathic pain, and cochlear implants for hearing loss. Notable examples still mainly in the research phase, include nerve-controlled prosthetic limbs, and retinal and vestibular prostheses. Lesser-known target organs include the stomach and bladder. These are smooth muscle-based electrogenic organs with dense neural innervation, where mapping of the electrical signals can hold useful diagnostic information. However, despite the recent surge of interest in optical techniques in electrophysiology, the translation of research laboratory innovations into clinical tools has barely started to materialize.

## III. OPTICAL TECHNOLOGIES IN ELECTROPHYSIOLOGY

The use of light for reporting electrical activity in living tissues was historically linked to visibly detecting an optical spark from electric eels and relating it to neurophysiology as demonstrated by John Walsh in a series of experiments in 1772–1775.^8^ Since then, the properties of light have been applied to the design of tools for electrophysiology with capabilities unattainable with conventional electrical systems. For example, light enables transmission of analog signals from many channels in a single optical fiber or in a single beam using wavelength division multiplexing or polarization-based multiplexing. This offers a huge advantage in terms of signal bandwidth and, thus, scaling to very large channel numbers.

It is important to consider both the fundamental properties of light and the optical properties of tissue when selecting an optical technique for a given electrophysiological application. The optical properties of tissues are mainly governed by the complex biochemical and physical structure of the tissue.^9^ Just like any substance, biological tissues can interact with light by means of absorption or scattering. Light absorption determines how deep an incident light beam can penetrate the tissue in a straight path, while light scattering describes the degree of deviation of the penetrating light from that path. Excitation and modulation of biological structures and molecules by light can be measured and used as a metric to provide functional information about the tissue. Light absorption by light-responsive proteins, ion channels, and advanced light-sensitive materials can be employed to generate thermal or electrical energy to stimulate cells and tissues.

The tissue response to light or vice versa (i.e., light modulation in response to tissue activity) is wavelength-dependent.^9^ Light at the visible spectrum (380–600 nm) normally has a low optical penetration depth in tissues owing to the high scattering of light. In the visible red and near infrared (NIR) spectrum (600–1000 nm), scattering in live tissues decreases and consequently penetration depth increases to a few millimeters. However, further in the infrared range (>1000 nm), optical penetration depth decreases back to sub-millimeters and can cause heating of biological tissues, due to the predominant absorption of mid-infrared light by water molecules. The light wavelength also defines the detection sensitivity in optical sensing and recording systems, regardless of whether the sensing element is a physical sensor or a light-sensitive fluorophore or biomaterial. Hence, the choice of wavelength in optical electrophysiology techniques relies on the application and the desired penetration depth within tissue layers.

Other important light parameters for optical electrophysiology tools include energy per pulse and energy per area of coverage (irradiance or intensity, W/m^2^), and polarization, i.e., the oscillation direction of light waves relative to the direction of light propagation.

In Secs. III A and III B, we identify established and novel, versatile optical technologies (both flexible and rigid), developed to advance the understanding and modulation of neural, cardiac, and muscle electrophysiology. These technologies include but are not limited to, calcium- and voltage-sensitive imaging using fluorophore dyes or genetic indicators, optogenetics, liquid-crystal electro-optical transducers, neuroplasmonics, organic and inorganic photostimulation, and more. We discuss the latest advances in these technologies aimed at improving the quality of measurements and efficiency of stimulation and highlight published applications. We classify optical technologies into two broad categories: recording and stimulation.

### A. Optical recording

#### 1. Voltage and calcium optical imaging

**FIG. 1.**
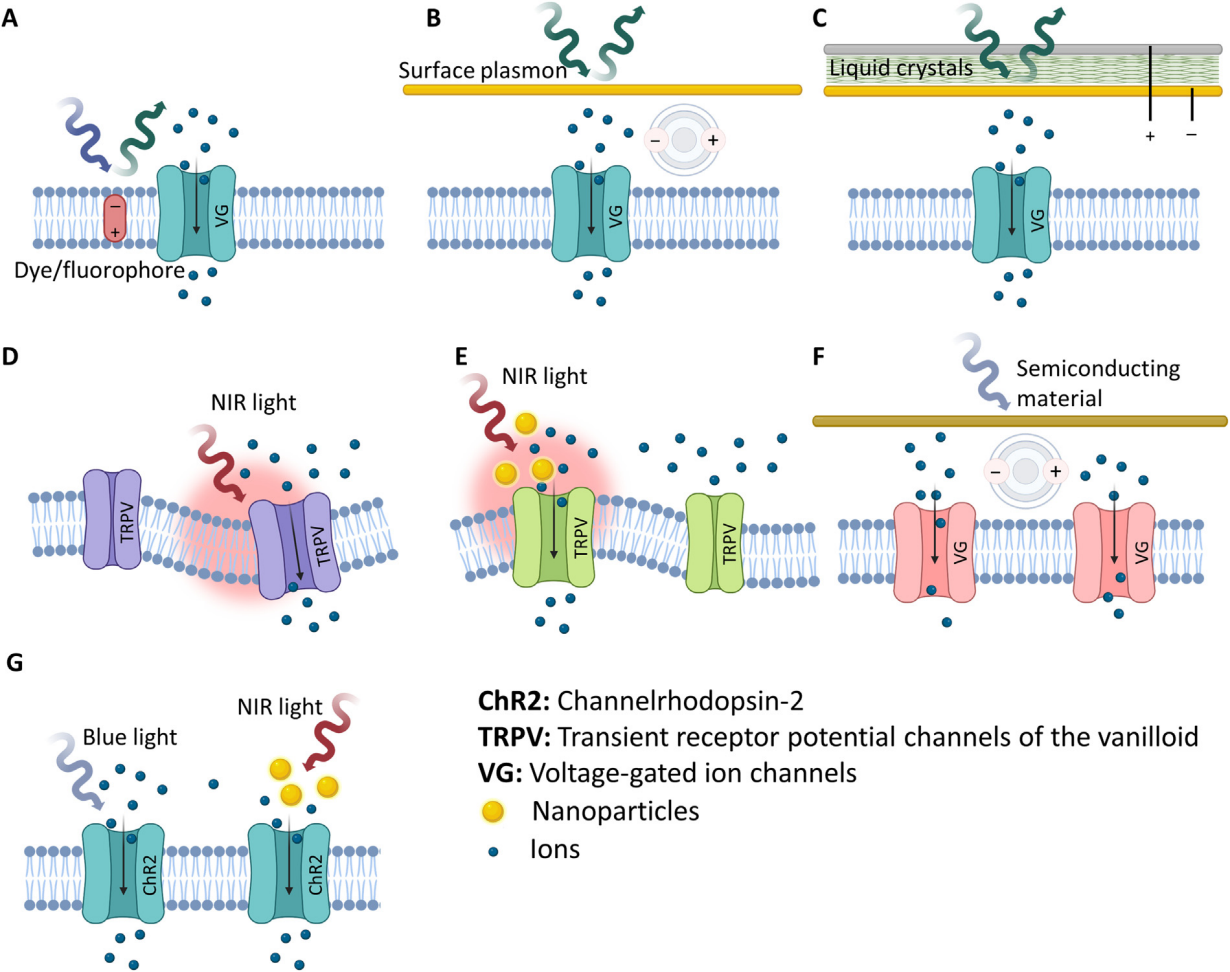
A schematic illustrating the mechanisms of optical modulation and sensing. (a) Voltage and calcium optical imaging is based on dyes or fluorophores changing their spectral properties in response to changes in cell membrane potential or intracellular calcium concentration. (b) In plasmonic recording, signals are detected via the surface plasmon resonance (SPR) phenomena, where small changes in the light properties are correlated with transmembrane potential. (c) Liquid crystals passively transduce electrical signals from cells into optical signals by changing the intensity of reflected light in response to variations in the sensed biopotential. (d) Photothermal stimulation, for example, using near-infrared light (NIR) is based on a localized heat source that triggers thermally mediated TRPV ion channels and/or induces changes in membrane capacitance. (e) Nanoparticle-assisted stimulation can be done through localized heating generated by illumination of nanoparticles embedded within tissues. (f) Organic and inorganic semiconducting materials, for example, photodiodes, can be used to mediate optical stimulation through transducing optical signals to electrical stimulation. (g) Optogenetic approaches include light-sensitive ion channels like ChR2 that are genetically expressed in the cell membrane and respond to direct wavelengths of light from a source or via nanoparticles providing wavelength upconversion. Created with BioRender.com.

Calcium- and voltage-sensitive imaging using genetic indicators or fluorophore dyes have become conventional tools for measuring real-time cellular electrophysiological activity from different spatial locations with a good temporal resolution. The dyes/indicators are sensitive to transmembrane voltage or intracellular Ca^+2^ concentration and, following excitation by light at a certain wavelength, emit light at a different wavelength in response to changes in transmembrane voltage or intracellular Ca^+2^ concentration [Fig. 1(a)]. From that, the genetically or chemically modified excitable cells are imaged using cameras or photodiode arrays, for mapping superficial regions, or confocal or two-photon microscopy for mapping deeper regions using a laser scanner and advanced photodetectors based on photomultiplier tubes. The excitation and emission spectra for common indicators are in the 430–700 nm range.^10^ There is a plethora of scientific literature on the recent advancements in this field, including membrane potential-dependent photoinduced electron transfer with fluorescent indicators^11^ and photoacoustic voltage-sensitive dyes of long wavelengths.^12^ With the high-speed measurements of the fluorescent signals, many *in vivo* studies have managed to image and report the dynamics of neural and myocardial activity in large populations of cells.^13,14^ The nature of this technology lends itself to multisite recording. In fact, the term “optrode” was originally coined to refer to optical fibers that transmitted the fluorescence signal from tissues to external photodetection systems. However, these methods are typically susceptible to different types of noise, such as hemodynamic and mechanical noises, as well as experiencing photobleaching and cell toxicity, which limits the procedure duration. In addition, signals are measured as relative changes in voltage or Ca^+2^ concentration rather than accurate quantification of the absolute transmembrane potential. More importantly, the type of excitation and detection tools and the current working principle as well as the efficacy and safety of fluorophores or genetic indicators hinder the translation of this technique into a flexible array design for chronic studies.

**FIG. 2.**
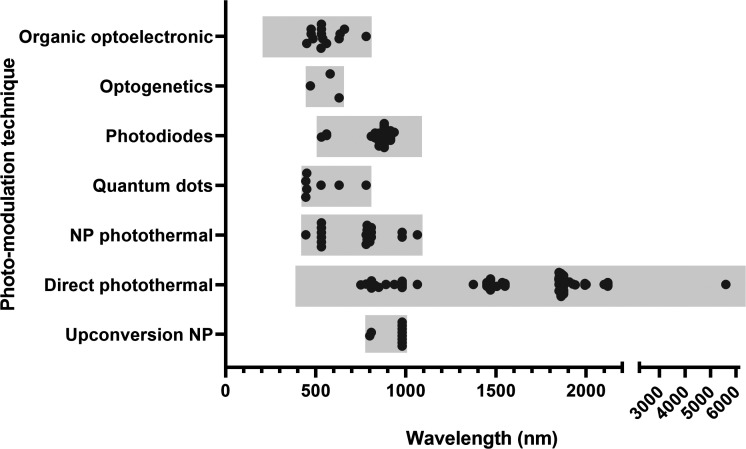
The range of wavelengths reported in reviewed studies utilizing different optical modulation (stimulation and or inhibition) techniques. NP: nanoparticle.

#### 2. Functional near-infrared spectroscopy (fNIRS)

Functional near-infrared spectroscopy (fNIRS) is a label-free imaging technique that detects small changes in light scattering or absorption caused by hemodynamic responses in the cellular volume, blood flow, and hemoglobin oxygenation due to the increased metabolic energy needs associated with an increase in the electrical activity of cells.^15–17^ This principle was first validated by detecting changes in membrane potential in cultured neurons through scattered light using dark-field microscopy.^18^ Then, noninvasive implementations of this technique advanced to employ optical fibers coupled with a light source, operating over the 650–1000 nm wavelength range, along with light detectors, which quantify the back-scattered light based on the penetration depth through tissue layers. Pioneering studies on the development of fNIRS technology started in 1992 with a single channel system and evolved into head-mounted multi-channel systems (10 or more detectors) starting from 1995.^19^

Advancements in the field of fNIRS imaging include increasing the spatial resolution with higher channel counts, incorporating light-weight fiber bundles, and developing portable and wireless multichannel systems.^20,21^ Despite the noninvasiveness, effectiveness, and adequate spatial resolution of fNIRS imaging, individual event analysis remains challenging owing to the significant temporal latency and poor signal-to-noise ratio (SNR).^20^

#### 3. Phase-sensitive plasmonics

Phase-sensitive plasmonics is one technique that has recently garnered special attention owing to its ability to provide label-free optical reporting of the extracellular electrophysiological activity of cells. The principle of its operation relies on the surface plasmon resonance (SPR) sensing phenomenon, which is dependent on the resonant oscillations at a metal–dielectric interface. The action potentials generated from the neurons and cardiac cells vary the refractive index of the interface. If a light beam is passed underneath or reflected off the underside of the interface, changes in the absorption/reflection of the light beam due to changes in the interface refractive index can be measured, creating the SPR signal [Fig. 1(b)]. For example, Kim *et al.* recorded SPR signals *in vivo* from rat brain and isolated rat sciatic nerves, demonstrating the detection capability of this technology.^22,23^

While SPR sensing has exhibited its effectiveness in neural recording, the measured signal quality and resolution are not yet comparable to traditional MEA recordings, and the low SNR remains a serious challenge. Moreover, there is no established mechanism to linearly correlate the SPR signals to the actual magnitude of the sensed electrophysiological signal. Recent progress includes the development of an array of plasmonic nano-antennas with enhanced cross sections, offering an ultrasensitive electric-field detection of about 10^2^–10^3^ V/cm in the extracellular environment.^24^ Array designs were achieved using plasmonic nanostructures, such as 3D gold nanopost-shells, nano-antennas, and nanoelectrodes to improve the spatial resolution of detection and flexibility introduced by integrating flexible patterned plasmonic substrates into the system, demonstrating bioelectrical recording from different tissue types [Fig. 4(a)].^25–27^

#### 4. Liquid crystal electro-optical transducers

A more recent label-free technology uses electro-optical transducers that feature light-modulated liquid crystals (LCs) to passively convert tissue biopotential signals into measurable optical signals.^28^ Representing the electrophysiological signals by LC-induced light modulation was first demonstrated in 1979 using nematic LCs coated on top of frog hearts.^29^ However, the recently developed LC electro-optical transducers incorporate deformed helix ferro-electric (DHF) LCs that are aligned in a pre-determined angular orientation inside a small device.^28^ More specifically, the LC material is sandwiched between two conductive substrates that create a potential difference across the LC layer whenever an electrical signal is applied. One of these conductive substances functions as a mirror, and the other is transparent allowing incident light from a light source to pass onto the mirror via the LCs. Light reflected off the mirror passes through the LCs again, then the transparent substrate before being transmitted to a photodetector [Fig. 1(c)]. The applied electric field changes the alignment of the LCs; and, the intensity of the reflected light passing through them changes accordingly. The direct correlation between the changes in the power of detected light and changes in the sensed voltage across the LC layer facilitates quantification of the biopotential signal. Al Abed *et al.* demonstrated detailed benchtop characterization and *ex vivo* and *in vivo* animal validation from cardiac tissue preparations and sciatic nerves, respectively, which provided insights into the recording capabilities and sensitivity of the DHF LC electro-optical transducers.^30^

In addition, preliminary results suggested that multi-channel LC arrays can be designed, fabricated, miniaturized, and scaled in dense configurations without electrode-amplifier impedance-related complications, overcoming a major limitation of conventional MEAs.^31^ However, further *in vivo* animal and *ex vivo tissue* testing is still required to assess the spatial-temporal resolution of the array system. While this sensing transducer is still at its embryonic stage, it is expected that the current rigid design will be transformed into a flexible interface by utilizing soft optical components to achieve arrays that can better match the compliance properties of excitable tissues and accommodate the natural motions and irregular topography of soft tissues.

### B. Optical stimulation

**FIG. 3.**
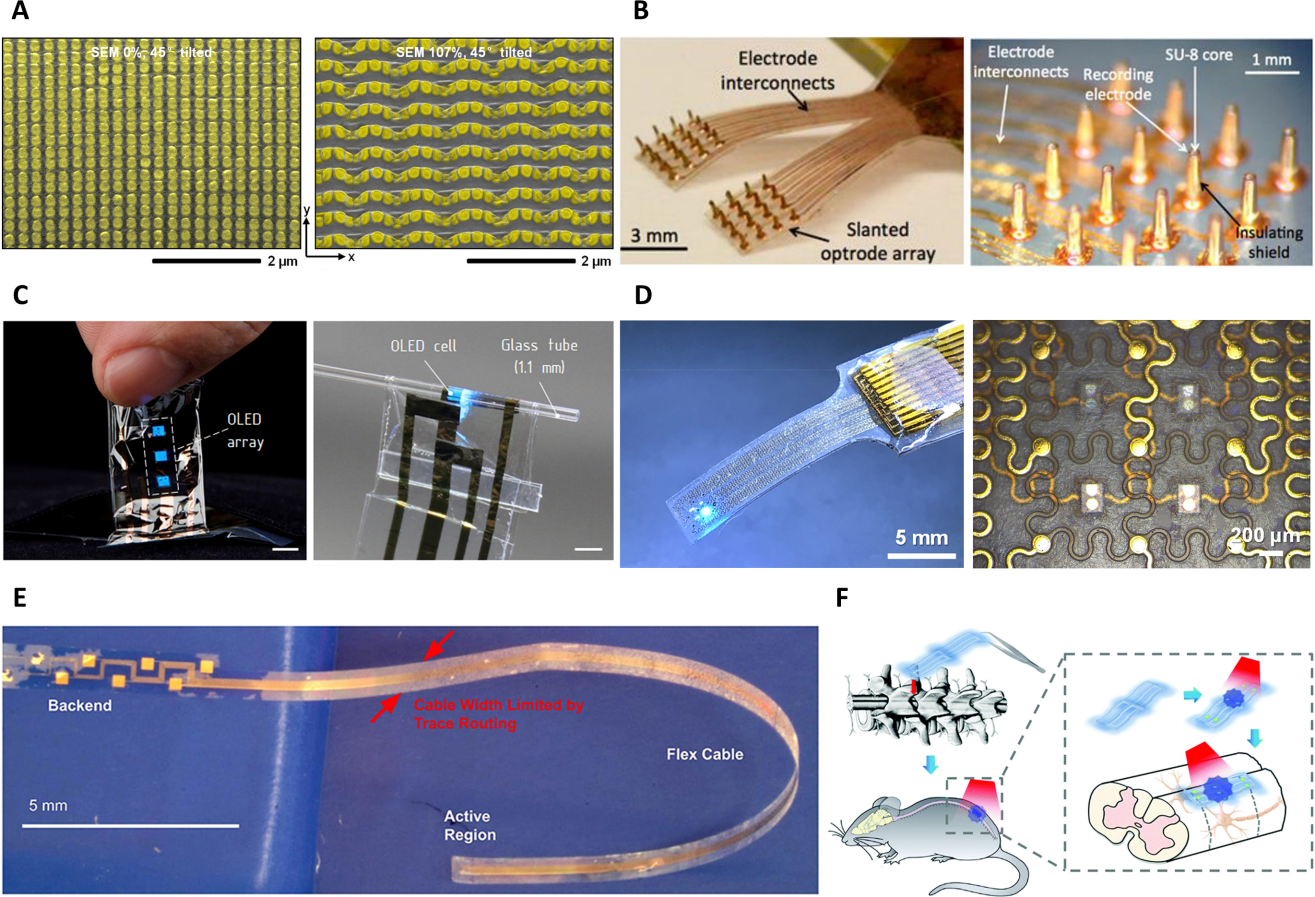
Flexible optrode devices. (a) Stretchable, dense arrays of plasmonic nanodisks for optical sensing. Adapted with permission from Gao *et al.*, ACS Nano. **9**(6), 5968–5975 (2015). Copyright 2015 American Chemical Society.^27^ (b) *μ*LEDs array with 32 channels, micro-waveguides, and microelectrodes integrated on a flexible parylene-C substrate for optogenetic stimulation. Adapted with permission from Kwon *et al.*, Front. Syst. Neurosci. **9**, 69 (2015). Copyright 2015 Authors, licensed under a Creative Commons Attribution (CC BY) license.^187^ (c) OLED array integrated into an ultrathin flexible parylene-C substrate for optogenetic stimulation. Adapted with permission from Dongmin *et al.* Proc. Natl. Acad. Sci. U. S. A. **117**, 21138 (2020). Copyright 2020 National Academy of Sciences. ^47^ (d) Flexible and stretchable array with serpentine-shaped interconnects for optogenetic neuromodulation. Adapted with permission from Ji *et al.*, Biosens. Bioelectron. **153**, 112009 (2020). Copyright 2020 Elsevier B.V.^44^ (e) Dense, double-sided, array of embedded *μ*LEDs onto flexible substrate for bi-directional optical stimulation. Adapted with permission from Reddy *et al.*, Front. Neurosci. **13**, 745 (2019). Copyright 2019 Authors, licensed under a Creative Commons Attribution (CC BY) license.^188^ (f) A flexible device with upconversion nanoparticles for optogenetic stimulation upon NIR triggering. Adapted with permission from Wang *et al.*, Nanoscale **12**, 2406–2414 (2020). Copyright 2020 Royal Society of Chemistry/Clearance Center, Inc.^67^

Light can be used to modulate biological tissue directly via photothermal stimulation and inhibition as well as indirectly via optogenetic techniques or using advanced organic and inorganic optoelectronic materials, and photothermal materials (Fig. 3).

#### 1. Optogenetics

Optogenetic technology forms a major area of research in the field of optical neuromodulation as researchers can accomplish gain or loss of function with a high spatial selectivity. This modality involves introducing genes that encode light-activated ion channels called opsins. The light-dependent activation of these channels elicits excitation or inhibition of excitable tissue [Fig. 1(g)]. In 1979, Francis Crick was the first scientist who emphasized the need to selectively control one specific cell type in neural tissue while leaving others unchanged, suggesting light as a method to achieve that.^32^ The insertion of exogenous opsins in neurons to modulate their activity was proposed in the early 2000s by Miesenböck.^33^ It was not long before other successful neuronal implantations of light-responsive proteins were reported, including the engineering of light-sensitive K^+^ channels in neurons.^34^

To achieve this type of modulation, a light source that spectrally matches the activation spectrum of the photosensitive opsin is required. Different wavelengths in the visible range are commonly used based on the type of the light-activated opsin, such as blue light (∼470 nm) for channelrhodopsin-2 (ChR2), yellow light (∼580) for halorhodopsin (NpHR), and red light (∼630 nm) for Volvox-Channelrhodopsin-1 (VChR1). The conventional approach for light delivery includes filtered light from an arc lamp or light-emitting diode (LED) coupled to an optical fiber. In the case of fiber guides, a single cannula can be used to inject the viral vector that expresses the opsin and direct the light to the targeted region. Numerous studies combined optogenetic control with transparent MEAs in densely packed designs to achieve bimodal devices. Advances include designing matrices of organic LEDs (OLEDs) for multisite activation,^35–37^ incorporating flexible polymeric connectors,^38^ incorporating bioresorbable optical waveguides,^39^ and improving wireless communication.^40,41^ More recent research and commercial efforts have focused on the fabrication of soft optogenetic arrays, capable of light delivery, for integration with tissues and organs. Such flexible designs have been effectively employed in the cochlea,^42,43^ brain,^44^ heart,^45^ and peripheral nerves.^46^ Furthermore, high density arrays of OLEDs were successfully fabricated for cell control using optogenetic techniques.^35,37,47^

Clinical translation of optogenetics for modulation of excitable tissue faces a number of significant hurdles. The safety of opsin genes and the long-term efficacy and safety of expressed light-sensitive ion channels must be demonstrated in humans. Techniques for localized delivery of these genes into target tissue, overcoming the limitations of current viral based delivery methods, require further development and clinical trials. More central to this review is the delivery of light. Progress is being made in terms of medical-grade optical fibers. An alternative, lesser-invasive approach that utilizes the tissue-penetrating properties of infrared light is the use of upconversion nanoparticles. This recent development has been extensively reviewed from many perspectives.^48–60^ These nanomaterials are capable of absorbing photons at the infrared range and converting their energy to emit photons in the lower visible wavelength regime required to activate photosensitive opsins [Fig. 1(g)]. The utility of upconversion nanoparticles for optogenetic modulation has been demonstrated in cultured hippocampal neurons,^61^ the brain,^62–66^ spinal cord,^67^ sciatic nerve,^68^ and heart.^69^

**FIG. 4.**
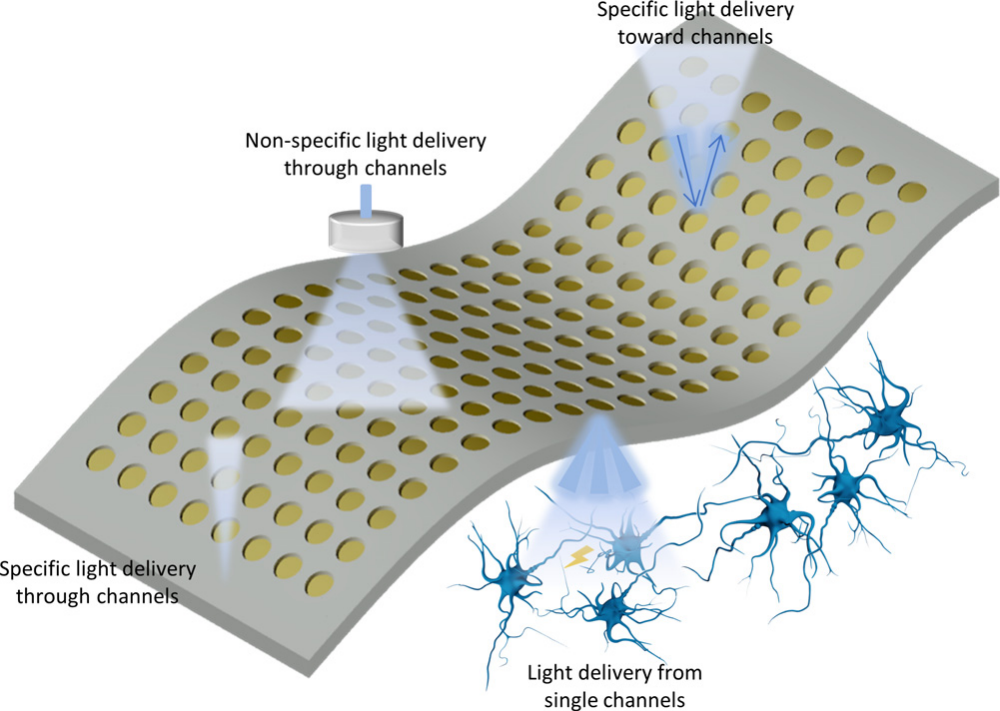
Modes of light delivery in optrode array devices. In optical stimulation, light can be either delivered nonspecifically through a transparent array towards the tissue or specifically through/from channels towards the tissue. In optical recording, both optical delivery and detection are done through specific channels.

Flexible upconversion devices were recently developed for optogenetic stimulation to enable soft integration and implantability in freely behaving animals [Fig. 4(f)].^67,68^ A notable recent development toward controlled light delivery was the fabrication of a multi-channel device by patterning a micropipette with pinholes coated with a number of nanoparticles, each with different excitation and emission properties, and capable of selective activation of optogenetic targets based on the timing and wavelength of the applied light.^64^

#### 2. Direct photothermal stimulation

The last 20 years have also seen the advent of light as a mechanism for cardiac and neural modulation (i.e., stimulation, inhibition, and pacing) without the requirement of introducing genes into cells. The photothermal mode of action can be achieved via direct infrared illumination of tissue,^70–75^ which will be discussed in this section, or indirectly via optical heating of materials embedded within the biological tissue (Sec. III B 3). These techniques overcome many of the limitations associated with optogenetics-based light stimulation, including the need to transfect cells with genes encoding light-sensitive opsins.^75,76^

Direct infrared modulation of biological samples has been demonstrated in cultured cardiomyocytes,^77–81^ cultured hippocampal neuronal cells,^82–85^ cultured cortical neuronal networks,^86–88^ cultured vestibular and spiral neurons,^89–94^ cultural retinal ganglion neurons,^90^ cultured cortical astrocytes,^95^ cultured neuroglioma cells,^96^ nerves and neuromuscular junctions of invertebrates,^97–103^ embryonic^104–107^ and adult hearts,^108,109^ sciatic,^110–116^ optic,^117^ cavernous,^118^ vagus,^119^ and dorsal root nerves,^120^ the cochlea,^121–147^ semicircular canals of the vestibular system,^148,149^ the retina,^150^ and brain^151–163^ and transcranially in humans.^164,165^ Applications include cardiac pacing, neural stimulation and inhibition (modulation), suppression of epileptic seizures,^162^ and high throughput screening of drugs for heart diseases.^81,166^ There have also been attempts at developing hybrid electrical-optical stimulation.^98,167^ A particularly noteworthy application, that has been the subject of concentrated research efforts, is the use of direct infrared light in the cochlea with the long-term aim of developing an optical cochlea prosthesis of a higher spatial resolution compared to existing clinical electrical prostheses, to restore hearing loss and enable finer auditory perception.^168^ The efficacy of NIR in stimulation of cochlea in healthy animal models has been demonstrated in a number of studies.^123^ However, translation of these findings to deafened animal models remains controversial with conflicting results reported by a number of research groups.^92,121,122,124,126,132,136,137,139,143^

There is consensus within the scientific community that light affects the cellular electrophysiology via a thermal action [Fig. 1(d)]. However, the exact mechanisms of how the light-induced temperature change modulates the biophysics of cells, to stimulate or inhibit action potentials, is still a subject of debate and ongoing research. Several hypotheses have been postulated including (1) altering the cell membrane capacitance via structural changes in the lipid membrane^96,169^ or heat absorption by water causing water movement on the micro-scale that alters the ionic composition of solution either side of the cell membrane,^101,170^ (2) formation of nanopores in the cell membrane,^82^ (3) activation of temperature sensitive ion channels in the cell membrane, the transient receptor potential channels of the vanilloid subtype (TRPV),^95^ (4) intracellular Ca^2+^ transients^163^ caused by release of Ca^2+^ from intracellular stores,^83^ and (5) modulation of intracellular calcium cycling^93^ with evidence implicating mitochondria as the primary organelle contributing to this release.^77,93^ (6) A modeling study of the effect of temperature on ion channel kinetics has suggested a major role of voltage-gated potassium channels,^171^ which was later confirmed by an experimental study.^100^ At a higher organ level, recent human studies suggest that pulsed application of NIR can modulate network oscillations in the human brain.^164,165^ These mechanisms arise due to either the spatial gradient and temporal rate of heating (change in capacitance^170^) or absolute temperature (TRPV^90^) subsequent to the illumination of cells with NIR light.

A broad range of wavelengths have been used (780–6100 nm), but most published studies applied infrared light (Fig. 2). The vast majority of published studies used single or multimodal optical fibers (<1 mm diameter) for delivery and conventional rectangular pulse waveforms, but other waveforms have been explored^147^ and were reported to improve performance efficacy including alternating, ramp-up or triangular. Several studies have demonstrated safety margins acutely in animals^119,129,172^ and humans,^120^ as well as chronically in animals.^116,142^ Progress is needed to advance the development of arrays with multi-channel addressing for multi-site NIR light stimulation. To the best of our knowledge, this has only been demonstrated in a fiber-optic array with temporal switching between channels,^161^ or as biophotomodulation head-mounted devices commercially available and exempt from regulatory approval as “general wellness devices.” Nevertheless, we do not see any major fabrication or material obstacles that could hinder the development of such arrays in the near future.^147^

#### 3. Indirect photothermal stimulation

Indirect photothermal activation works by using light to illuminate materials embedded in tissue. The absorption of photons by these materials leads to an increase in their temperature, which in turn can exert similar effects on tissue as direct light stimulation, albeit with higher intensity more focused thermal changes [Fig. 1(e)].

Nanoparticles can mediate photothermal stimulation of excitable tissue^168,173–176^ and have been applied to modulate cultured hippocampal neuronal cells,^177–181^ cultured rat dorsal root ganglia neurons,^178,182^ cultured rat auditory neurons,^183^ cultured cardiomyocytes,^177,184,185^ cultured spiral ganglion cells,^176^ brain slices,^186^ the cochlea,^141^ sciatic nerve,^176,189,190^ heart,^191^ and leg.^182^ Nanoparticles have also been reported to inhibit neuronal activity in a light intensity dependent manner.^180^

Hybrid approaches combining electrical stimulation and nanoparticle-mediated photothermal stimulation has also been proposed and tested in an *in vitro* model.^192^

Photothermal stimulation is usually combined with electrical recording to simultaneously sense the signals elicited by cells. Nanoparticles have been used to coat a nanoelectrode^184^ and microelectrode arrays,^181,193^ enabling photothermal modulation of cells a technique that can be applied in the future in high-resolution arrays and to the end of an optical fiber,^179^ providing an *in vitro* proof-of-concept for future *in vivo* endoscopic or penetrating optrode array applications. By combining quantum dots (see Sec. III B 4) and nanoparticles into a layered device, it was possible to produce a single channel device capable of generating either positive or negative photocurrents, resulting in cell hyperpolarization or depolarization and action potential generation.^194^

Key challenges that need to be managed for clinical translation are cytotoxicity, localized delivery of nanoparticles to the target sites, and investigations into the long-term effects of chronic implantation of these constructs.

#### 4. Quantum dots for optoelectronic stimulation

Quantum dots can be used to fabricate light-sensitive heterostructures that can generate electrical currents upon illumination by light.^195^ In a recent development, Jalali *et al.* reported a quantum funnel structured from a rainbow of quantum dots; the optical energy of the illuminating light is transferred along the excitation gap gradients between sequential quantum dots until this energy is finally captured by the largest quantum dot and converted to electrical energy for neuronal stimulation.^196^ Photostimulation via quantum dots has been applied in neuroblastoma cell cultures,^196^ PC-12 Ad cell cultures,^197^ and cultured rat hippocampal neurons.^198,199^ Like nanoparticles, localized delivery and cytotoxicity and pre-clinical safety studies need to be demonstrated before human trials.

#### 5. Stimulating optoelectronic photodiodes

Biomedical photovoltaic cells are primarily based on photodiodes and utilize the same technology employed in solar cells by harvesting light energy and converting it to electrical stimuli to excite biological tissues [Fig. 1(f)]. Additional considerations are imposed by the biological application and clinical translation including biocompatibility, miniaturization, development of array embodiments for multi-site stimulation, and ensuring efficient energy transform to elicit responses from cells without the need for high energy light application that could thermally damage cells.

The current–voltage (IV) curve of photodiodes depends on the illumination power. The circuit is considered open when no current flows but a voltage is outputted, and it is considered closed circuit when current flows but no voltage is outputted. The maximum voltage and current values depend on the load (resistance) of the tissue and can be increased by adding photodiodes serially. However, this slows down the discharge of the photodiodes after the termination of light illumination driving the electrical stimulus. This can be overcome by placing shunt resistors between the photodiodes to accelerate the discharge.^200,201^ Biphasic waveforms can be achieved by biasing the photodiodes with a biphasic power supply.^202^ However, because of the discharge nature of photodiodes, symmetrical biphasic current waveforms cannot be achieved, which may have longer-term safety implications.

Applications include targeting the sciatic nerve as a form of functional stimulation to induce muscle contractions,^203,204^ retinal vision prostheses that have been trialled preclinically^202,205–233^ and clinically,^234–237^ floating light-activated microelectrical stimulator for the spinal cord^238^ and cortex,^239^ and heart pacing.^203^ Established micro-fabrication processes for photodiodes have enabled the realization of arrays for multi-site stimulation, in both rigid and more recently soft flexible variants. For example, preliminary studies demonstrated the viability of flexible organic photodiodes (OPD) arrays for high-resolution retinal prostheses.^240^

Recent advances in the technology include photodiodes arranged in a 3D honeycomb array configuration with a return electrode elevated vertically relative to and surrounding each active electrode to enable selective stimulation of cells,^241^ photodiodes based on nanowires, which enable tens of semiconductor p–n junctions to be vertically stacked,^225,226^ and fabrication of photoelectrochemical cells using heterojunctions of non-porous hard and nanoporous junctions as an alternative to conventional p–n junctions.^203^ Optoelectronic photodiodes also have been combined with LC-based biopotential sensing elements as proof-of-concept bidirectional light-utilizing devices and demonstrated to record from and stimulate the sciatic nerve *in vivo.*^242^

#### 6. Conjugated polymers for optoelectronic stimulation

Conjugated polymers are currently being assessed as alternative materials for silicon-based semiconductors towards the design of soft and flexible optoelectronic devices.^243^ Conjugated polymers are organic materials that exhibit an extended π-electron system due to the alternating single and double bonds in their chemical structure. The small bandgap between the π-bonding and π*-antibonding orbitals underlies their photophysical properties; typically, this bandgap has a range of 1–4 eV,^244^ overlapping with the ultraviolet-visible (UV-VIS) or NIR optical domains. This characteristic inspired their use first in organic photovoltaic solar cells^245^ and later as photoactive materials for the modulation of electrophysiological signals in living cells upon light illumination.^246^ Coupled with their high absorption coefficient, conjugated polymers are emerging as next generation photoactive materials for biomedical applications,^247,248^ such as photomodulating human stem cells^249^ and restoring light sensitivity in blind retinas.^250^

Interfacing the organic optoelectronic device with living cells or biological tissues will require the device to operate in an aqueous electrolyte. Several mechanisms have been elucidated at the interface between the device and the electrolyte upon light illumination. These include photothermal, photocapacitive, photofaradaic, and photochemical reactions.^246–248^ The type of organic semiconductor, the light intensity and duration, and the device architecture are all parameters that influence which mechanism occurs at the material/electrolyte interface, with reports of more than one mechanism co-existing in the same system.^248^

The photothermal process occurs when the absorbed light is converted into heat. This process is triggered when the light is illuminated for long durations (milliseconds–seconds) or in response to high intensity light (>10 mW mm^−2^) pulsed at a shorter timescale (in the range of few milliseconds or less). The physiological response is attributed to the same processes underlying the response to direct photothermal stimulation (discussed in Sec. III B 2).

Light absorption by the semiconductor generates an exciton that separates into a hole and an electron given the appropriate device architecture.^247,248^ A photocapacitive process occurs if charges accumulate at the device/electrolyte interface, forming a double layer. The current generated by this process is a transient ionic current that persists in the electrolyte until charge neutrality is reached at the polymer/electrolyte interface. On the other hand, the process is photofaradaic if a charge is transferred and reacts with molecules such as oxygen in the electrolyte solution.^251^ Here, the charges do not accumulate at the interface, and thereby, a steady constant current is generated and maintained as long as the diffusion of the acceptor molecule is not hindered. Photocapacitive and photofaradaic processes co-exist; however, which process is dominant depends on the type of semiconductor and device architecture.

Photochemical reactions occur when both electrons and holes are transferred into the electrolyte solution resulting in oxidation and reduction reactions. These reactions happen when the organic semiconductor is illuminated with high intensity light for extended exposure times. Products produced from these processes have been shown to modulate the electrophysiological signal; however, they have also been associated with toxicity and degradation of the organic semiconductor. Similarly, the photofaradaic process produces reactive oxygen species^252^ that are associated with photocytoxicity when produced in high concentrations.^253^ Subsequently, the photofaradaic mechanism is less favored in the stimulation of biological tissues.^248^

Regardless of the process for cellular excitation, further evaluation of organic optoelectronic materials is required to characterize the relationship between their surface area, light power, and current or voltage thresholds required to modulate excitable tissues. This knowledge will contribute to setting the minimum size of channels when designing and fabricating organic optoelectronic arrays.

## IV. DESIGNS OF FLEXIBLE OPTRODE ARRAYS

In this section, we provide an overview of the architectures and designs used for soft and flexible optrode array interfaces and devices in the electrophysiology context, including planar, surface conforming, penetrating, and endoscopic or catheter-based.

### A. Multisite and multi-layer tissue arrays

**FIG. 5.**
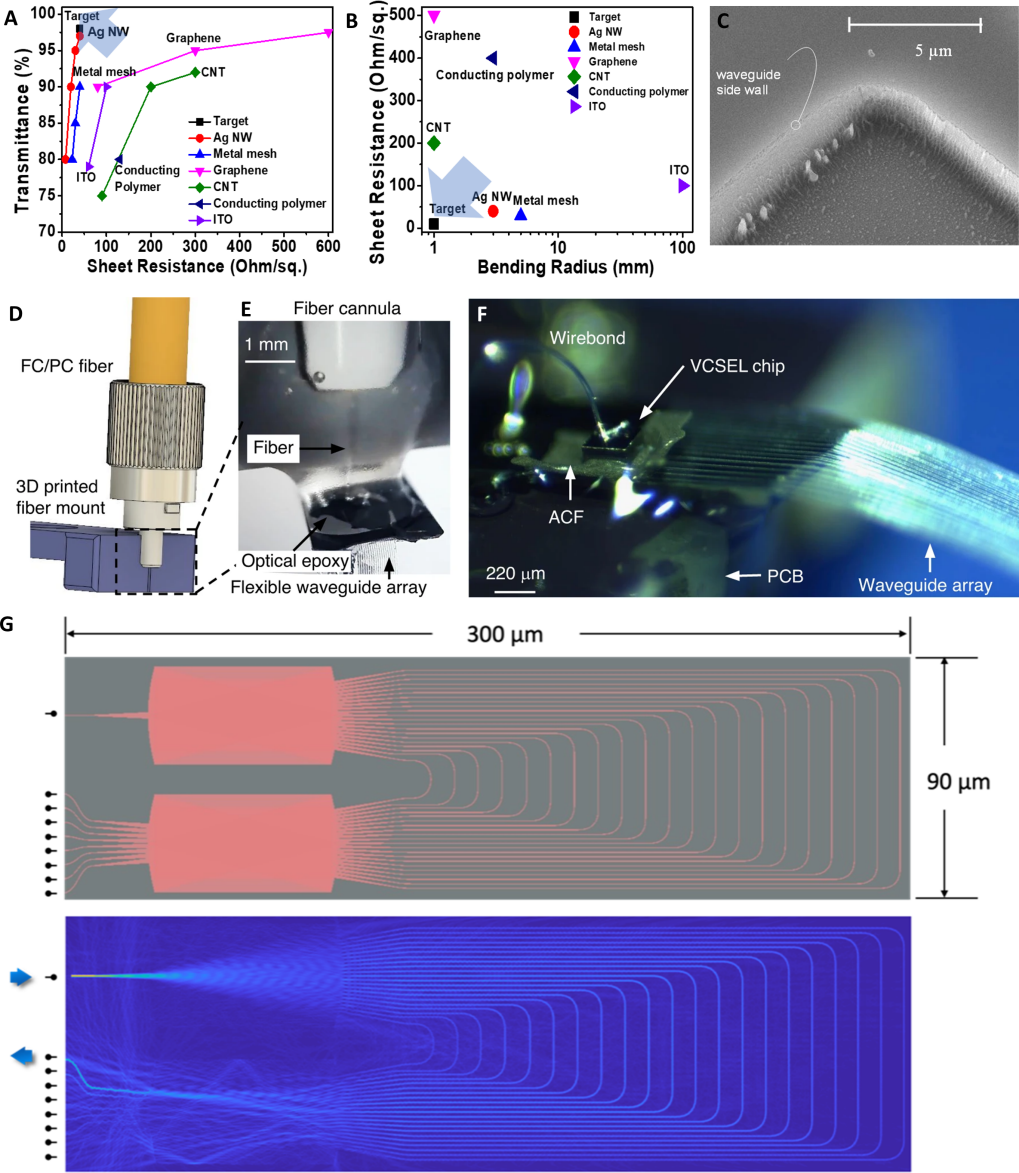
Design considerations for flexible optrode arrays. (a) Electro-optical properties and (b) electromechanical properties of transparent conductive films/electrodes in flexible applications. Adapted with permission from Sohn *et al.*, Materials **12**, 2526 (2019). Copyright 2019 Authors, licensed under a Creative Commons Attribution (CC BY) license.^285^ (c) Side-wall roughness in integrated optics waveguides. This picture of a 90° bend in diamond waveguides clearly shows the roughness present on the sidewall due to the etching process step of its fabrication. (d) Schematic of a fiber-bonding 3D printed mount featuring a V-groove for fiber alignment.^38^ (e) Optical fiber bonded to a flexible parylene waveguide using optical epoxy.^38^ (f) A vertical-cavity surface-emitting laser (VCSEL) chip bonded to the input port of a flexible parylene waveguide using anisotropic conductive film (ACF).^38^ (d)–(f) Adapted with permission from Reddy *et al.*, Microsyst. Nanoeng. **6**, 85 (2020). Copyright 2020 Authors, licensed under a Creative Commons Attribution (CC BY) license. (g) Compact arrayed waveguide grating. The top panel illustrates a top view of an AWG implemented in silicon (core) and silicon dioxide (cladding) to operate around 1535 nm and supporting eight channels with a channel separation of 2.5 nm. The bottom panel illustrates the routing of a specific input wavelength (1527.5 nm) into a specific output channel.

Enabling simultaneous control or monitoring of the spatiotemporal patterns of neural and cardiac activities can be achieved through multisite and multilayer arrays. There are currently two designs of optrode arrays in electrophysiology applications, regardless of whether electrode channels are present or not. The first configuration includes arranged channels with an external light delivery onto the substrates for optical interrogation, where light interfaces directly with the device but not the biological tissue. While this layout has been achieved in phase-sensitive plasmonics,^26,27^ liquid crystal electro-optical transducers,^30,31^ and nanomaterial-assisted photothermal stimulators,^193^ only the former technology has been implemented in a flexible array design at this stage. The second configuration involves multiple optical sites that deliver light from the device directly to the tissue.^44,254^ Example applications include optogenetics and direct photothermal stimulation. Many applications like fNIR, optogenetics, and photothermal stimulation combine both light delivery and readout from tissue. However, transparent MEAs in combination with nonspecific trans-array light delivery for tissue visualization, optogenetics stimulation, or fluorescent imaging is beyond the scope of this review.^255,256^ Figure 5 illustrates the different modes of light delivery in optrode arrays.

Delivering light from the device to the tissue involves optical structures and components, such as LEDs and waveguides that need to comply with the flexible array design.^38^ In recent years, advanced photonic and optic structures have played important roles in the functionality and design of both surface conforming and penetrating optrode arrays. Microfabrication techniques have enabled coupling arrays of *μ*LED chips to 3D needle-shaped waveguides to penetrate tissue and deliver light simultaneously to deeper subregions of neurons in the brain [Fig. 4(b)], leaving most of the flexible device on the organ surface.^187,257^

The density and arrangement of optical input/output ports or *μ*LEDs along the probe shank in a similar configuration to that of Michigan and Utah arrays provide access to deeper tissue layers.^258–261^ Similarly, planar and surface-type optrode array designs can involve contiguous illumination sites on a surface conforming backbone to provide access to curvilinear surfaces of organs, such as the heart and brain regions in fissures.^47,262^ Some studies employed dense *μ*LEDs with the ability to control and activate some or all of the *μ*LEDs according to the region of interest.^36,37^ While the overall structure of planar arrays does not allow physical penetration through the tissue, using a certain wavelength range can provide deeper activation and control of deeper tissue layers, like the case of using red light for optogenetic excitation. Most of the aforementioned efforts have been targeted towards brain applications.

In the research laboratory realm, fluorescence imaging probes based on flexible optical fibers, endoscopes, or catheters have been developed for over two decades for optical imaging of calcium- and voltage-sensitive fluorophores. This technique has been more recently formalized as fiber photometry^263^ and has historically progressed from epifluoroscent to confocal and multiphoton imaging.^264–268^ These systems deliver optical pulses to target tissue to excite a fluorophore of interest, and the emitted light is collected and transmitted by either the same or a secondary optical fiber.

Cardiac optogenetics is an evolving area,^269^ but flexible catheter or endoscope technology for delivering light to either the epicardial or endocardial surfaces of the heart or intramurally has not caught up with the progress in molecular biology techniques and hence is a major hurdle in clinical translation of such technology. Toward this end, Klimas and Entcheva proposed technical design considerations for the development of endoscopic systems for simultaneous cardiac optogenetic manipulation and optical imaging, taking advantage of access routes for routine catheter-based clinical imaging procedures in cardiology (e.g., angiography, optical coherence tomography, and light spectroscopy). The proposed system is based on conventional microscopy setups for fluoroscopy imaging and optogenetics excitation, but with the objectives replaced with a fiber coupler that couples the light beam to an optical fiber (bundle) for catheter-based access to cardiac structures and a GRadient Index (GRIN) lens for focusing light from the optic fiber onto the tissue and collection of fluorescent light from the tissue.^270^

### B. Multifunctional arrays

The rapid development of miniaturized sensors, MEAs, and light-based technologies in parallel with advancements in microfabrication techniques has naturally progressed research efforts toward combining different functionalities into a single flexible multimodal device.^271^ Examination of the scientific literature, focusing on devices with at least one optical functionality, revealed advanced and ingenious material manipulation and fabrication processes to generate both surface conforming and penetrating miniature multi-functional devices. These devices feature electrophysiology recording and stimulation capabilities, combined in some cases with temperature, pH, strain sensing, and microfluidics for drug delivery (Table I). It is worth noting that in terms of the optical capabilities, these devices have been limited to the integration of *μ*LEDs and waveguides or optical fibers for optogenetics (often just a handful). Incorporation of waveguides for NIR stimulation, inorganic photodiodes, organic optoelectronic or photothermal materials into multi-modal devices will be an exciting future development in the field.

**TABLE I.** Examples of multifunctional flexible or soft arrays featuring at least one optical component. Rigid arrays are considered out of scope. S: surface, P: penetrating, MEA: multi-electrode array, WG: waveguide or optical fiber, *μ*LED: micro light emitting diode, DD: drug delivery, SG: strain gauge, +: 1 channel, ++: 2–9 channels, +++: 10–99 channels, and NA channel count details not available.

Structure	Recording MEA	Stimulating MEA	WG	*μ*LED	pH	Temp	DD	SG	Reference
S	+++			++	+++	+++		+++	272
S	+++			++					273
S		+++		++					274
P	NA			NA					188
P	+++		+				+		275

## V. FABRICATION TECHNIQUES TO ACHIEVE FLEXIBLE OPTRODE ARRAYS

Optrode arrays are heterogenous structures typically fabricated from a combination of dielectric, conductive, optoelectronic, and light generation and waveguiding components. We investigate the key challenges posed by designing flexible optrode arrays by exploring the class of materials used for each array component and mapping these to different applications and targeted tissues. For each component, we discuss the relevant state-of-the-art fabrication techniques for achieving soft and flexible structures. We conclude with an overview of emerging light-modulated materials that are expected to be embedded into optrode arrays in the near future.

### A. Dielectric structures

These components encompass the substrates, encapsulation, or insulation layers found in optrode arrays. Substrates are the underlying layer of the array that hold all the components together, while encapsulations are the protective layers that minimize failure modes of implants and prevent waste and materials from moving between the tissue and the device, reducing host response. Both the substrate and encapsulation of the array normally form the main interfacial material with the biological environment; hence, assessing their impact on the biocompatibility of the device is a crucial aspect, which will be discussed thoroughly in Sec. VI G.

Polymers are the most common class of materials used in numerous flexible implants as substrates, insulation, encapsulation, or a combination of all owing to their biological performance, mechanical flexibility, and ease of tuning their properties. It has been shown that a wide range of synthetic polymers function well in applications that require long-term stability in hostile surroundings and exhibit little response to implantation.^276^ An earlier review paper extensively discussed the biological effects of multiple flexible polymeric biomaterials for medical implants, including cytotoxicity, sensitization, and irritation.^277^ Synthetic polymers such as polyimide (PI), parylene, polydimethylsiloxane (PDMS), polyurethane (PU), polyethylene (PE), and SU-8 are widely used in implantable devices, neuro-prosthetics, and cardiac catheters.^277^ These polymers meet key physical characteristics required in such common devices, including excellent insulation properties, high thermal resistivity, and superior mechanical stability. Generally, the transformation of these polymers from a powder or liquid form to thin films simply involves a few fabrication steps that include physical or chemical deposition and heat treatments.

The mechanical behavior of such polymers can be tailored using chemical and physical techniques. Making use of this characteristic, combining synthetic polymers with other materials, such as organic nanomaterials and hydrogels, as composites can enhance flexibility and provide a softer contact to the overall system. In addition, while polymeric materials mostly exhibit a high elastic modulus (∼GPa) compared to that of soft tissues (∼kPa), their stiffness and flexural rigidity can be massively reduced by decreasing their thickness and fabricating ultra-thin films, which enables conformable lamination to curvilinear surfaces of organs, such as skin, heart, and brain.^278^

### B. Conductive structures

Arrays combining optical and electrical functionalities consist of electrically conductive components that transfer electrical signals from excitable tissues to a certain part of the interfacing device or to an external outlet or from external controllers to stimulating electrodes or LEDs. Transparent electrodes, conductive layers, interconnectors, and wiring are among their features. The mechanical flexibility of these materials remains a concern, especially when relatively thick layers are required to attain both high conductivity and acceptable optical transmittance.^279^ This is because the underlying physics governing their performance characteristics results in a rather simple trade-off between the electrical and the optical properties.

Transparent, electrically conductive materials are commonly prepared from semiconducting oxides of indium, tin, zinc, and cadmium. Indium tin oxide (ITO) and zinc oxide (ZnO) are some traditional materials that have been used in optical devices for electrophysiological applications^280,281^ owing to their high conductivity and transparency. However, even thin layers of such materials are prone to deformation, delamination, and cracks when deposited onto flexible substrates and components, resulting in an electrical failure in chronic and even in acute applications.^282^

**FIG. 6.**
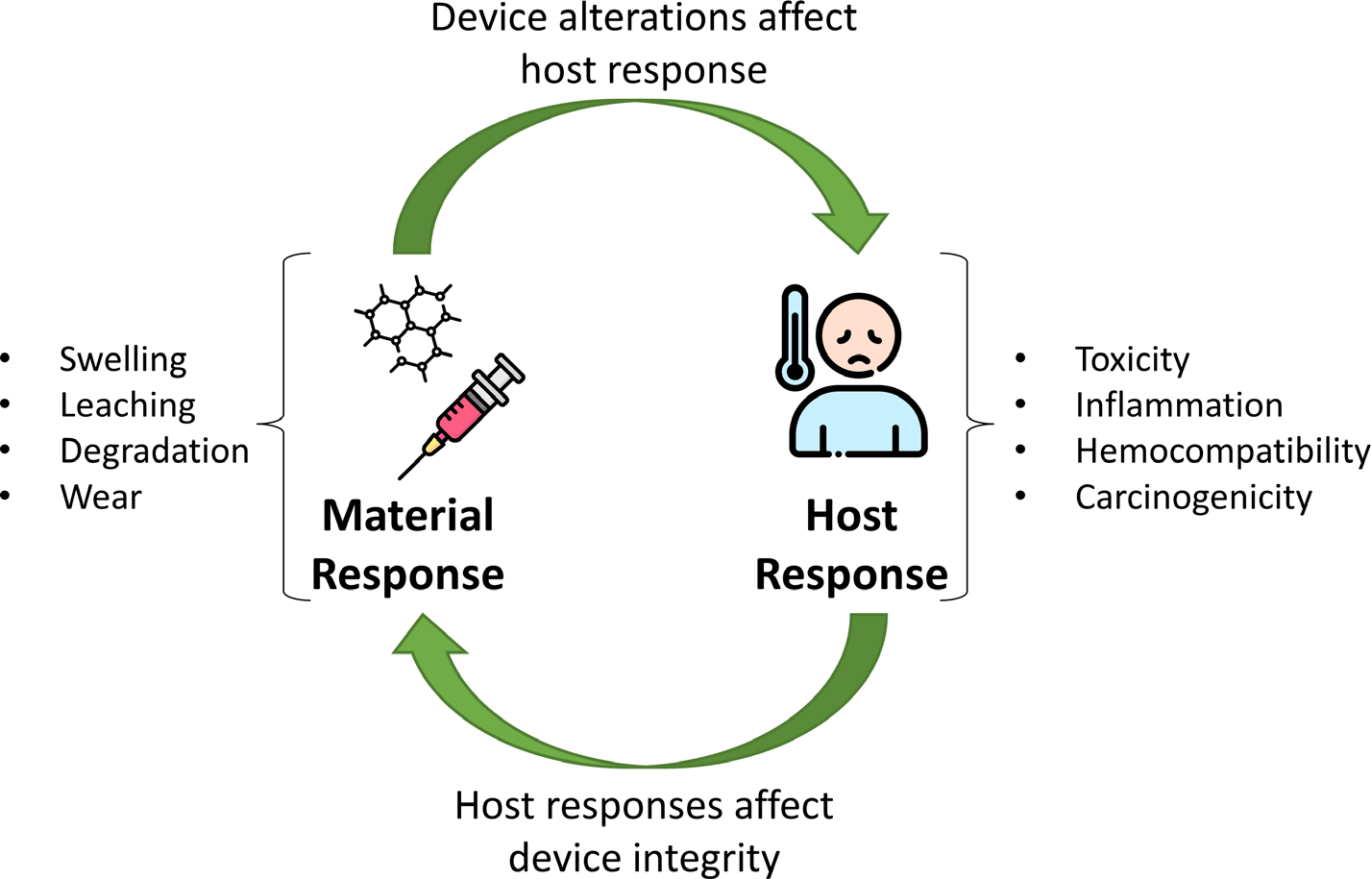
Critical considerations in evaluating safety risks of medical devices include the material and host responses and interactions between these aspects. Examples of material responses that may occur are shown as are the major categories of host responses; these examples are not exhaustive.

Such brittle materials can be replaced with other electrically conductive materials that are mechanically flexible. Some examples include conjugated polymers, carbon materials (i.e., single or few layers of graphene and its derivatives), and 2D transition metal dichalcogenides. Transition to soft variants requires considering factors when fabricating flexible conductive components. Although electrical conductivity is the key focus, conformity, ease of fabrication, and biocompatibility are also critical considerations. In flexible optrode arrays specifically, these materials have to sustain their conductivity while being mechanically stable as well as optically transparent. Different forms of tests have been conducted to examine the electro-mechanical stability of transparent conducting materials including cyclic stretching, bending, and mandrel bending.^2,283,284^ Figures 6(a) and 6(b) illustrates the electro-mechanical and opto-electric properties of some flexible and rigid transparent conductive electrodes/films.^285^

Conjugated polymers, such as poly(3,4-ethylenedioxythiophene) polystyrene sulfonate (PEDOT:PSS), polyaniline (PANi), polypyrrole (PPy), and their derivatives are shown to exhibit excellent mechanical stability and low resistivity.^2,286–288^ Carbon materials, including graphene^256,289^ and carbon nanotubes (CNT),^290–292^ are also widely used as conductive electrodes and layers in numerous biomedical and industrial applications. This is due to their remarkable characteristics, such as high mechanical and chemical stability and large aspect ratio.^293^ Creating hybrid mixtures of carbon materials and conjugated polymers is a common approach to improve the device/tissue interface and enhance its mechanical stability.^294^ Biswas *et al.* demonstrated how the inconsistent transmittance and poor mechanical flexibility of traditional transparent conductive films, such as ITO, can be solved by replacing them with a hybrid of polymer and carbon materials.^295^ Recent studies reported the fabrication of graphene-based array devices with optical transmission of >90% throughout a broad range of wavelengths, being used for optogenetic measurements.^256,296,297^

There are several means of fabricating and depositing transparent conductive layers onto flexible optoelectronic devices. While solution-processing, evaporation, spin coating, and sputtering are among the most straightforward methods of deposition, more advanced and automated methods are required to achieve better controllability in terms of feature size, thickness, and material properties. Creating metal gratings of nanostructures was achieved using printing technologies, such as jet printing, screen printing, and nanoimprint lithography, to act as transparent conductive electrodes in optoelectronic devices.^298,299^ These films are densely perforated with periodic, nanoscale mesh or spheres on a substrate using metals of a high work function like Au and Ag, which makes the overall film semitransparent. This technique was used in OLEDs, organic solar cells, and MEAs that combine both optical and electrical measurements.^300–302^

Overall, recent advances indicate that many organic and inorganic materials could be utilized as conductive components in flexible optrode arrays with the right materials, design, and methodological choices and illustrate relatively higher performance, particularly in the long term, compared to rigid arrays.^303^

### C. Optoelectronic structures

Device architecture is a key design factor to consider when developing optrode arrays with semiconductor based optoelectronic components. The application of conjugated polymers as an optoelectronic interface for light-mediated stimulation is enabled by their organic nature. Conjugated polymer substrates can be made flexible, soft, and stretchable.^304^ Their ease of processing facilitates fabrication of light-weight optoelectronics that can be easily made into implantable medical devices.^250,305^

Conjugated polymers have been used either as a pristine photoactive material without any additives or has been processed into a photovoltaic device. Examples of conjugated polymers investigated as photoactive materials for biological applications include poly(3-hexyl thiophene) (P3HT), poly(*p*-phenylene vinylene) (PPV), polythiophene derivatives, and polyfluorene (PF), with P3HT being the gold standard photoactive material for bioelectronic devices.^246^ Thin films of pristine conjugate polymers have been fabricated on non-conductive^249^ and conductive substrates.^250^ In a typical photovoltaic device, the *p*-type conjugated polymer is used as the donor material combined with *n*-type material that is an acceptor. The photovoltaic device can be either made of a bilayer heterojunction or bulk heterojunction. In a typical bilayer heterojunction, the conjugated polymer is spun-coated onto a conductive substrate such as ITO. A hole transport layer might be applied on the ITO substrate before the deposition of the photoactive polymer. On top of the conjugated polymer, a layer of the acceptor material is deposited, which might be followed by the deposition of an electron transport layer and a conductor. The bulk heterojunction device has similar components except that the donor and acceptor materials are blended to maximize the interface between the two phases in comparison to the planar junction obtained in the bilayer architecture.

In a simplified device architecture, the photoactive conjugated polymer has also been used as the photovoltaic material without the need of a substrate or an acceptor material. The most prominent example being P3HT nanoparticles.^306–308^ The light-evoked stimulation observed following the injection of the P3HT nanoparticles in blind retina was attributed to the photocapacitive mechanism.^307^ Similarly, a scaffold fabricated from electrospun polycaprolactone-P3HT (PCL-P3HT) fibers and coated with PPy has been shown to be effective in optically stimulating PC-12 cells enhancing neurogenesis.^309^ The authors attributed the photoeffect to either a capacitive and/or faradaic mechanism with the need for further studies to delineate between the two mechanisms. Of note, they showed that the conjugated PPy aided in scavenging reactive oxygen and nitrogen species, that otherwise were persistent in uncoated PCL-P3HT scaffolds. This study provides a new design consideration to overcome challenges related to the photochemical effect associated with the application of the photoactive conjugated polymer.

So far, organic semiconductor optoelectronic structures have been demonstrated in single unit/channel devices. Their transition into arrays can borrow from inorganic semiconductor optoelectronic arrays composed of photodiode channels providing photovoltaic stimulation. These photodiodes are typically fabricated as heterogenous structures using inorganic materials, such as crystalline (c-Si) silicon or hydrogenated amorphous (a-Si:H), or organic materials, such as poly[[2,5-bis(2-hexyldecyl)-2,3,5,6-tetrahydro-3,6-dioxopyrrolo[3,4-c]pyrrole-1,4-diyl]-alt-[2,2′:5′,2′′-terthiophene]-5,5′′-diyl] (PDPP3T), phenyl-C61-butyric acid methyl ester (PC61BM), as well as merocyanine–rhodamine.^240,310^ When an incoming photon of light with sufficient energy strikes a photodiode, it creates mobile electron–hole pairs (*p*–*n* junctions), producing a photocurrent and leading to charge accumulation around the photovoltaic cell.^200,201^ This charge is delivered to tissues via electrodes. These inorganic optoelectronic arrays have been under development for over two decades and have been trialed in humans and offer learnings that can be adapted when fabricating organic optoelectronic devices. For example, stacked structures can be fabricated whereby several *p–n* junctions are serially connected by tunnel junctions to increase the energy harvesting from light and, hence, output voltage levels for tissue stimulation. A variety of fabrication techniques can be employed to achieve a variety of electrode configuration and stimulation patterns. Reported interface configurations in inorganic optoelectronic arrays include a pair of electrodes acting as anode and cathode,^204^ a shank with anodic and cathodic contact surfaces,^205,238,239,311^ arrays of monopolar electrodes with a proximal^207–210^ or distal^225,226,233^ common return, arrays of bipolar electrodes (each active electrode is surrounded by a return electrode, but all return electrodes are connected),^202,212–223,227,228,230,232,234–236^ or classical bipolar configurations.^224,231^

Adaptation and merger of device design and fabrication techniques from inorganic photodiodes arrays with the development of photoactive material systems based on conjugated polymers could drive the advancement of soft and flexible optoelectronic arrays.

### D. Light-generating structures

LEDs, like photodiodes, are semiconductor-based components that behave through the interaction of light energy with their material system and have broad application for light stimulation in electrophysiology. They primarily consist of two semiconductor substrates of opposite charges; a *p*-type (doped with an electron acceptor) and an *n*-type (doped with an electron donor), and an undoped (pure) intrinsic semiconductor is placed in between. The semiconductor material and bias mode determine whether this structure will act as an LED or as a photodiode. More specifically, for an LED when current passes through this structure in a way that forward biases the junction, light emission occurs at a certain wavelength according to the bandgap of the material used. This light is then delivered to either the tissue or to an interfacing device.

Micro-LEDs (*μ*LEDs) have opened new avenues for completely untethered fiberless array-based systems. This is because optical fibers can physically constrain natural movement and limit long-term studies in freely behaving subjects as they tether the light source to the implant during the entire experiment. The latest developments in *μ*LEDs focused on reducing their size, lowering power consumption and heat dissipation, as well as optimizing output power, making them an essential element for the fabrication of optical interfaces especially in optogenetic applications. Integrating the LEDs onto the electrical interconnects on the chip or substrate surface can be done simply using monolithic microfabrication techniques, transfer techniques with a flip-chip bonder, or manual assembly using a conductive epoxy or paste.

The placement of individually addressable *μ*LEDs in the form of an array with a certain pitch enables precise control of light delivery with high spatial resolution, directing research efforts toward miniaturization of LEDs down to a typical neuronal or muscle cell size.^37^ More importantly, integrating *μ*LEDs onto a flexible substrate with appropriate spacing can create a multichannel LED array that is overall flexible even if the *μ*LEDs *per se* are made of stiff materials [Fig. 4(e)].^188^ Stretchability can be also attained by incorporating serpentine-shaped metal interconnects between each LED element to achieve an entirely stretchable array [Fig. 4(d)].^44,45,312^

Semiconductors often employ many inorganic materials, including but not limited to silicon (Si), gallium arsenide phosphide (GaAsP), indium gallium arsenide (InGaAs), and gallium phosphide (GaP). Of these, gallium-nitride (GaN) on Si or sapphire substrates is the most common inorganic material used for blue LEDs, as this wavelength overlaps with the absorption band of some fluorescent tags used for neural imaging, as well as the ChR2 channelrhodopsin used in optogenetics. While blue *μ*LEDs are extensively used in optogenetic-based optoelectronics,^4^ the recent trend of developing red-shifted opsins has led to a growing interest in fabricating red *μ*LED arrays using aluminum gallium indium phosphide (AlGaInP).^313^

Inorganic materials and components are usually encapsulated with flexible polymers to reduce mechanical damage and create a soft interface to tissues. However, recent trends in semiconductors manufacturing are shifting toward employing organic materials, owing to their light weight, mechanical flexibility, and low toxicity.^314^ There are many examples of organic materials, such as triphenylamine derivatives (TPD), oxadiazole derivatives (PBD), and distylyl derivatives for OLEDs [Fig. 4(c)].^47^

All in all, given that most semiconductor microelectronic components are usually cumbersome and have poor biological performance, compact encapsulation with coatings or materials with acceptable biocompatibility is a critical step in the fabrication process prior to implantation.^315^

### E. Optical waveguides

Optrode arrays with stimulation and recording functionalities demand optical materials that enable flexible and effective light delivery over a desired length toward targeted tissues and cells. While conventional glass optical fibers (i.e., silica optical fibers) are suitable for light delivery, the continuous development of bio-integrated devices such as implantable optrode arrays requires even softer waveguides exhibiting specific biological performance and physical characteristics. As a result, a variety of transparent bioinert materials, like polymeric biomaterials, have emerged as alternatives. Examples of conventional optical polymers include PDMS, parylene C, SU-8, poly(methyl methacrylate) (PMMA), polyurethane (PU), and polycarbonate (PC).^316^ Choosing the suitable candidate material for the waveguide design depends on the desired length and application, as well as the sources of optical loss intrinsic to the material itself, which will be explained in Sec. VI A. More importantly, selecting a polymer material of biocompatible properties, like PDMS and parylene C, is crucial for flexible implantable optrode arrays.^38^

The optical and mechanical properties of flexible polymers make them good candidates for creating bendable and/or stretchable optical waveguides. The fabrication of such flexible waveguides can involve one or more of the following processes: material deposition, photolithography patterning, plasma polymerization, thermal drawing, and 2D–3D printing. As an example, Canales *et al.* employed a thermal drawing process (TDP) to generate long, flexible, high throughput, and well aligned fiber probes that enable simultaneous optical readout and optogenetic stimulation.^317^ Through this process, a single polymer or combination of polymers such as PC, carbon black doped conductive polyethylene (CPE), and cyclic olefin copolymer (COC) are processed at a very high melting-temperature to create thin probes of micrometer scale diameters. The high flexibility of those probes permits them to adapt with anatomical changes during motion with minimal optical loss of 1.6–2.6 dB cm^−1^.^317^ Another noteworthy advancement is the fabrication of stretchable photonic devices using epoxy polymer materials and chalcogenide glass, which were monolithically integrated on an elastic PDMS substrate with serpentine-shaped design to enable stretchability.^318^

### F. Emerging light-modulated materials

Other classes of light-activated components have been reported in optrode devices. These novelty materials include photothermal nanoparticles for indirect light stimulation, upconversion nanoparticles for optogenetics, optoelectronic quantum dots, and LCs for passive electro-optical transduction of biopotential signals.

Photothermal nanoparticles can take the form of spheres,^186^ rods,^176,179,183,189,190^ stars,^180^ hexagons,^191^ or horns.^182^ The majority of published studies to date have used gold^176,179,181,183–186,189–192^ nanoparticles. Other materials include carbon,^141,182,319^ mercury telluride (HgTi),^320^ and polydopamine,^177,186^ with reports of chemical modification to improve efficacy by coating gold nanoparticles with PEGylated polydopamine and conjugation with an anti-TRPV1 antibody to allow association with temperature-sensitive TRPV channels in the cell membrane.^186^ Alternatively, gold nanoparticles were conjugated with PEG-cholesterol to facilitate binding with the lipid cell plasma membrane.^178^

Upconversion nanoparticles for optogenetic stimulation have been demonstrated in animal studies as implantable single channel devices packaged within glass microneedle tips,^63,65,66^ encapsulated within parylene C cylinders^321^ on a dendrite-like-gold-inverse-opaline-film,^68^ or with polydimethylsiloxane,^69^ poly(methyl methacrylate) polymer,^61^ or polypropylene^67^ backbones.

Common quantum dots that have been studied in biomedical research are based on indium,^197^ indium phosphide/zinc sulfide (InP/ZnS),^194^ or lead sulfide combined with P3HT-PCBM^199^ or P3HT^198^ to form heterojunctions. The use of quantum dots as optoelectronic materials is still in its infancy compared to semiconductor photodiodes or even conjugated polymer based optoelectronic materials, and its fabrication in array configuration is yet to be demonstrated.

Electro-optical transducers based on DHF-LCs is an emerging light-modulated component for sensing of biopotential. These transducers can be fabricated either in a single channel device format with bipolar or monopolar connections to tissue or as multi-channel arrays. Optimizing the alignment of LCs is tied to improved sensor sensitivity. This is achieved by sandwiching the LCs between two alignment layers via capillary action. Even though only a rigid array has been reported, the current material choice for the alignment layers is rubbed PI and, hence, is appropriate for future development of soft and flexible variants of these devices.

## VI. CHALLENGES IN TRANSFORMING RIGID ARRAYS INTO SOFT VARIANTS

In this section, we explore the design considerations, technical requirements, and difficulties encountered when manufacturing flexible optrode arrays made of soft/flexible components to interface intimately with biological tissues in acute and chronic settings. We base this analysis on market evaluation of the current needs and potential clinical applications.^322^

The progress of increasing the spatial resolution of diagnostic systems and therapeutic prostheses has been incremental. We foresee that optical technologies have an opportunity to enable orders of magnitude improvement in these systems. For sensing arrays, this will enable more precise diagnosis of electrical disorders in the brain and heart. Current neuroprostheses on the market or under clinical trials have a very low number of electrode channels relative to the sensory encoding capability of the sensory end organs, for example, the human cochlea. In this instance, there are physical problems of how to generate electrical fields that can reach target neurons while only selectively targeting neurons encoding a narrow range of sound frequencies, as is required for refined auditory perception. The ability of light to stimulate a focused spot will enable higher channel count and higher resolution implants that will overcome the limitations preventing high-resolution variants and finer auditory perception (e.g., for the case of an optical cochlea implant, listening to music, conversation in a noisy environment).

Our survey of the literature has identified that not all optical technologies are at the same stage in terms of becoming arrayed devices. We do not see any fundamental obstacles to the development of flexible arrayed versions of single channel optrode devices. Comprehensive characterization of the light density and intensity required to activate light-sensitive materials will aid in determining the minimum size of each channel and tissue interfaces in these arrays. This could potentially suggest the need to improve materials or their chemical modification to enable array channel sizes equivalent to the state-of-the-art MEAs. Characterization and comparison of performance of arrays under stationary and flexible conditions (e.g., cyclic bending) is of paramount importance for validation of prototypes prior to further development into preclinical and clinical devices.

We have found that the vast majority of reviewed optrode arrays are yet to realize the full potential of light as an information carrier. Optical multiplexing and guidance of light to enable addressing of selective channels in an array using photonic circuits is a major advantage for optrode arrays that remains untapped. Once realized, we envision optical electrophysiological systems with orders of magnitude higher number of channels compared to conventional MEAs. To enable this advancement, photonic principles and techniques need to be incorporated into the design of arrays while fabricating under the constraint of material systems that are soft and appropriate for use in biomedical devices. Toward this goal, we explore several key challenges that if met could enable optrode arrays to match and surpass the performance of research and clinical MEAs.

### A. Mechanical compliance while minimizing optical losses

The development of high-resolution, high channel count optrode arrays for electrophysiology will eventually require the integration of tens of thousands of optical elements onto a flexible substrate. These elements include waveguides, light-modulating liquid crystals, micro-mirrors, refracting plasmonic surfaces, and lenses for collimation or diffusion of light generated by LEDs or delivered by the waveguides. Irrespective of the precise choice of such substrates, the question remains as to how to distribute the light effectively over the entire area of the array and read back the optical signals generated by the array.

The integrated optical circuitry necessary to distribute and collect light is subject to numerous design constraints—for example, control of polarization and elimination of unwanted cavity resonances—but at its core, the issue is one of optical losses. The main sources of optical losses are (1) absorption and scattering of light due to intrinsic material properties; (2) scattering of light due to surface roughness; and (3) leakage of light due to waveguide bending.

The first mechanism is intrinsic in nature and, consequently, once the material system is chosen, is independent of mechanical compliance. It is nevertheless of fundamental importance to choose the right system as it impacts on numerous aspects of the device performance, its integration with light sources and detectors, choice of wavelengths and coupling to the outside world (a.k.a. butt-coupling).

Surface roughness on the other hand is not intrinsic to the material systems but rather depends on the fabrication process. Small random deviations from perfect linearity of the waveguide sidewalls [Fig. 6(c)] are what explains the five orders of magnitude greater loss in optical circuitry (typically dB/cm) compared to that experienced in optical fibers (typically dB/km).^323^

It can be derived formally that loss due to random surface roughness is maximal when its correlation length $L_{c}$ is comparable to the beating length $2\pi /(\beta -kn_{cl})$ between the fundamental guided mode and the forward-directed radiation field.^323^ Here, we have introduced β as the fundamental mode propagation constant, k the light wavenumber and $n_{cl}$ the cladding index. For a typical waveguide, this occurs for values of $L_{c}\approx 100\;\mu m$ or lower. Since conformability should only introduce deviation from perfect linearity on the scale of millimeters or centimeters, it should not induce any additional loss from this perspective.

Mechanical compliance inevitably introduces bending, which leads to light leakage by coupling to the (non-guided) radiation field. Bending loss (as it is known) follows the Beer–Lambert law $P(z)=P(0)e^{-\alpha z}$, where P(z) represent the guided power at position z along the waveguide, and α is the attenuation coefficient due to the bending loss.

What is noticeable about that nature of the attenuation coefficient α is its dependence on the radius of curvature $R_{c}$ is itself of exponential nature: $\alpha =c_{\alpha }\;\text{exp}(-c_{R}R_{c})$, where $c_{\alpha }$ and $c_{R}$ are constants.^323^ Hence, small changes in the radius of curvature can lead to catastrophic leakage. In fact, it can be argued on the basis of its exponential dependence on bend radius that shrinking the size of an optical circuit by a given factor *q,* while keeping the waveguide geometry constant, results in an exponential increase in the overall bending loss. Clearly, the material system, loosely referred here as the flexible substrate, hosting the optical circuitry, must be chosen to support a given density of optical components, and straying from that density must take into account this exponential dependence. Also, care must be taken not to reduce bend radii below an agreed, pre-calculated value, for example, ∼1 mm for silica-on-silicon waveguides or ∼5 μm for silicon-on-insulator waveguides.^324^

This highlights the importance of choosing an appropriate material system as flexible substrates in order to accommodate multiple optical components at high density while minimizing bending and scattering losses to optimize the performance of optical circuitry under physiological stresses and strains. Conformability-introduces bends, not in the place of the optical circuit, but perpendicular to it as it conforms (bends) to the surface of a biological tissue. The radii of curvature of such bends are unlikely to be on the order of millimeters or below; hence, a well-chosen material system should not exhibit important bend loss due to conformability.

### B. Connecting optical devices to waveguides

Optically connecting photonic devices to the outside world requires mode matching. In essence, this means that the spatial distribution of the light coming from the outside world, most likely from optical fibers, matches that supported by the photonics circuit's waveguides. A mismatch between mode distributions inevitably leads to what is known as coupling loss.

The importance (and impact) of this mode mismatch—akin to impedance mismatch in the electronics world—depends on the nature of the material system used to implement the on-chip optical circuitry. For example, silica-on-silicon waveguides^323^ can be made to be well matched to standard optical fiber modal distribution. Such is not the case for silicon-on-insulators,^325^ and countermeasures must be put in place.

A number of approaches have been developed over decades to overcome mode mismatch, these include: adiabatic mode converters, resonant coupling, grating assisted coupling, and multimode coupling.^326,327^ Each has its pros and cons, fabrication issues, and specific performance when expressed in terms of coupling efficiency typically expressed in dB loss per connection.

The additional difficulty introduced here is caused by the requirements of flexibility. Presumably, a soft and conformable substrate supporting the optical circuitry would be difficult to (mechanically) connect reliably to optical fibers. One solution would be to design fiber arrays such as those used to connect fiber ribbons to integrated optics chips. These would include an area where the flexible substrate could be solidly attached after ensuring that mode coupling is optimized. For example, Reddy *et al.* connected compact vertical-cavity surface-emitting laser (VCSEL) chips to the input of a flexible parylene-based waveguide array using micromirrors for input coupling and bonded this connection by an anisotropic conductive film (ACF) [Figs. 6(d)–5(f)].^38^

### C. Flexible and soft photonic circuitry for passive multiplexing

The addressing of a specific optical or optoelectronic channel in a given optrode array can be achieved passively using an approach developed for the telecom industry, namely, wavelength division multiplexing.^328^ The basic idea is to subdivide the arrays into groups of channels and then assign a specific wavelength to each channel within a group. Using this approach, it is then possible to use a passive multiplexer such as arrayed waveguide gratings [Fig. 6(g)] to route a specific wavelength to its assigned channel.

When using wavelength for addressing via multiplexing, there is also needs for simple(r) filters that can be realized using Bragg gratings, which are periodic structures inscribed within guiding structures.^329^ Such filters can be used to isolate a specific wavelength—either to add or remove it from a given channel—or to couple light in and out of the plane of the optical circuitry.^330^

Both arrayed waveguide gratings and Bragg gratings rely on light interference via the precise control of accumulated phases to achieve their stated goals (multiplexing/demultiplexing or add/drop functionalities) and thus are very sensitive to both (1) induced stress and strain and (2) induced stretch by the flexibility requirements.

For example, if we consider a simple stop band filter implemented in a single-mode waveguide^331^ using a BG, it is possible to show that the central blocked (Bragg-) wavelength is given by $\lambda_{B}=2n_{e}\Lambda$, where Λ is the grating period, and $n_{e}$ the fundamental mode effective index. Obviously, any stretch of the grating will change its period Λ and, thus, results in a commensurate shift of the Bragg wavelength.

The effect of stress and strain is more subtle and manifests itself through its influence on the effective mode index $n_{e}$. In general, stress and strain will induce birefringence in the guiding structure through the photoelastic effect (a.k.a. stress-optic effect),^332^ which can make an otherwise isotropic material birefringent (i.e., optically anisotropic). The net effect is to change the nature of the effective index $n_{e}$ from scalar to tensorial, and, thus, dependent on the polarization of the light.

If the implementation of a multiplexing scheme relies on the precise control of the polarization (it need not be), flexibility requirements may have deleterious effects that need to be addressed. If not, the change in the effective index $n_{e}$ induced by stress and strain would nevertheless change the accumulated phases of the propagating light with potentially consequential impact.

A number of approaches can be taken to ameliorate the situation,^221^ starting with an appropriate choice of material systems with intrinsically low stress-optic coefficients. Furthermore, waveguide cross sections can be designed to impose polarization direction to the optical fields in order to counterbalance stress-induced polarization effects.

### D. Transdermal and wireless optical transmission

A main driver for the development of optrode arrays worldwide is their foreseeable application as chronic implants for preclinical studies in the electrophysiology research field and as clinical therapeutic devices. When interfacing optrode arrays with excitable tissues, two key engineering aspects have to be considered: (1) how data flowing to, from, or to/from the implant will be managed and (2) how the associated system will be powered. As for traditional biomedical implants such as pacemakers or cochlear implants, the volume of any technology required by the neural interface support system will, in general, be too large to be placed at the tissue interface site given constraints imposed by the biological tissue and surgical procedures. As such an implanted system would be split into a neural or cardiac interface sub-system and a support sub-system as is traditional.^333^ Such a split also adds flexibility to the system design since each sub-system can be better optimized and has indeed been applied in most recent optical implanted systems.^41,334–337^ In the case of electrophysiology interfaces with multiple contact points (arrays), a requirement for simultaneous control and individual addressing of the contact points would be assumed. The most straight-forward approach to achieve this is by including suitable electronic interfaces in the support sub-system, such as microcontrollers for controlling individual elements of the array (e.g. *μ*LEDs).^338^

Some implanted systems, as for instance traditional pacemakers, may be self-contained without requirement to external (non-implanted) support systems. Powering of such devices require an implanted battery or, if the power draw is sufficiently low, can be powered by an implanted energy scavenging system.^339^ In the more general case, however, implanted systems need to communicate with external devices and often have power draw requiring power being supplied from an external source. A requirement for both transdermal power transfer and bi-directional transdermal data transfer would generally be expected in implanted optrode array systems.

The assumed presence of electronics in the support sub-system leaves the system design fairly agnostic to the technology used for both transdermal power and data transfer as well as the method used to connect the implant with the support sub-system. The implanted electronics allow for implementation of a large variety of power converters, data encoding, and sensor/actuator interfaces, as can be seen in recent literature. Most sub-system interconnects use electrical wires^337^ although optical fibers are also seen.^334^ For power transfer, conventional magnetic field coupling^336^ is common but both photovoltaic arrays,^41^ and radio wave power absorption (RFID)^335^ are seen. Data transmission is likewise found using traditional magnetic coupling,^333^ radios,^335^ or light transmission,^41^ which are the most suitable methods for transdermal power transfer, and communication is highly dependent on the particular requirements for each optrode implant system.

### E. Thermal and chemical compatibility

The distinct advantage of fabricating rigid optrode arrays for electrophysiology is that the range of substrate materials most typically used (silicon, glass, fused silica amongst others) are processed using well established and proven micro- and nano-fabrication techniques. Additionally, inorganic semiconductors (Si, GaAs, and GaN) are well known for their suitability to enabling high-performance optoelectronic and electronic circuits. In this device context, common fabrication processes include photolithography and associated wet processes, metal/dielectric/transparent conductive oxide (TCO) deposition by means of thermal or e-beam evaporation, magnetron sputtering or chemical vapor deposition (potentially plasma assisted), subtractive wet and dry (plasma-based) etches, and many other thermal or wet processes. Translating such arrays from a rigid form-factor to a flexible architecture is necessary for reasons that have already been discussed, but this fundamental architectural change brings with it distinct fabrication-related challenges.

To ensure appropriate levels of flexibility, polymer layers are typically spun or cast onto a suitable rigid carrier substrate enabling subsequent fabrication steps to occur with minimal handling difficulties. In order to later “release” the flexible substrate from its carrier, a sacrificial layer is usually present.^340^ Polyvinyl alcohol (PVA),^341^ polystyrene (PS),^342^ and metals, such as aluminum,^254^ are typical materials used for this purpose. The idea is to eventually dissolve away the sacrificial layer using, for example, water (PVA), toluene (PS), HCl (Al), or acetic acid (Mg). Sacrificial layers must thus be chosen carefully to avoid early release issues due to process steps requiring wet processing such as etching of ITO using HCl, which might adversely affect an aluminum sacrificial layer. In a similar vein, unwanted surface modification can occur on substrates or deposited materials particularly due to prolonged exposure to acidic release agents. It is thus important to select both the sacrificial layer and release agent carefully.

Related to the issue of chemical compatibility, optical biomedical devices utilizing materials such as liquid crystals or nanowires can be particularly sensitive to chemical exposure. In the case of liquid crystals, solvents such as acetone or isopropyl alcohol can be particularly detrimental to the functioning of the active material. Mitigations include paying careful attention to provision of protective layers and encapsulation.

Thermal considerations also come into play where annealing steps are required. Curing or cross-linking polymers to improve strength/flexibility/optical properties can result in detrimental effects if, for instance, large thermal expansion coefficient (TEC) mismatches are present. This results in structural weakness, cracks, and device quality issues. Devices with active materials, such as liquid crystals or nanowires, can also suffer detrimental effects from excess thermal heating. Nanowires can undergo thermal deformation affecting their functional efficiency^343^ and liquid crystal devices can easily undergo phase transitions, which lead to loss of liquid crystal alignment and thus device failure.

These are all important factors to consider carefully during the design phase of the device so that through careful planning, the many process steps can be executed successfully and with high yield.

Beyond device fabrication, thermal and chemical compatibility should also be considered to ensure device stability during the operation of the optrode arrays. Materials that are exposed to the external environment need to be chemically resistant to various sterilization processes and biological fluids as will be discussed in Sec. VI G. The operation of optrode arrays is expected to generate heat due to propagation of light within the arrays or generation of light. The photothermal effects on the array's constituent materials, especially the thermal tolerances of polymers, should be analyzed and mitigated if found to be detrimental. Optrode arrays that warrant particular attention in this regard are those with direct and indirect thermal stimulation functionality or novelty optoelectronic arrays, where the opto-electric conversion efficiency is initially unknown, and a relatively large light intensity might be required for tissue activation, which could potentially lead to thermal-induced material damage.

### F. Manufacturing scale-up

Optrode arrays are complex devices combining electrical and photonic components integrated on flexible substrates appropriate for biological applications. Their fabrication processes involve multiple, non-automated steps and is currently done manually in a cleanroom environment. The complexity of these processes depends on the components involved, which are unique or not normally encountered in conventional MEA fabrication. These challenges will be exacerbated when manufacturing scale-up is required.

One of the major hurdles in selecting a fabrication system to manufacture optrode arrays is the use of numerous different substances being constructed in an automated single process. Optrode arrays require meticulous positioning of transparent and conductive substances, light-activated and photonic components, and additional optical connectors in different layers. The compatibility of materials (especially soft materials) with each other and with fabrication processes, the ability to deposit them at the same time, and the incorporation of novel elements into the manufacturing process all require unconventional thinking.

Additive manufacturing has emerged as a promising complementary fabrication technology for the production of optical as well as electrical components in electrophysiology devices. The ability to accurately lay out multiple materials with a high resolution and precision is vital for the fabrication of any multi-layered interface. When constructing multilayer devices with multiple materials, the need for post-processing steps to ensure structural integrity and reduced surface roughness brings challenges and opportunities to advance 3D printing technologies.

Two-photon polymerization and inkjet printing are some 3D printing examples used for transparent optoelectronics. Two-photon polymerization has been proven to print shapes with sub micrometer precision for 3D hybrid structures using a mixture of polymers and nanoparticles. However, the system's compatibility with different ranges of materials and fabrication speeds are still restricted.^344^ Inkjet printing offers the potential for large-scale, batch processing outputs and provides the flexibility to print a variety of materials counting dielectrics, conductors, and active materials on a variety of substrates such organic and flexible substrates, plastic and papers, in addition to the conventional silicon and glass wafers. This allows for rapid and cost-effective prototyping where, for instance, resistors and conductors can be manufactured at room temperature simultaneously with reduced material waste.^345–347^

An example of such printing technologies is reported by Athanasiadis *et al.* for the fabrication of a multifunctional membrane with embedded electrical, optical, and fluidic components on a flexible silicone carrier substrate.^348^ Different printing systems were utilized, such as micro-extrusion and inkjet printing, to fabricate and combine these components into a single device using different types of transparent silicone elastomers as the main flexible material. The fabricated membranes were hundreds of micrometers in size and successfully stimulated and recorded optogenetic hiPSC-derived cardiomyocytes. The study found that while 3D printing offers unique advantages, such as ease of prototyping and the ability to create complex geometries, challenges in achieving miniaturized features, high-resolution deposition, and surface smoothness remain persistent.^348^

The ability to manufacture optrode arrays using rapid, technology-driven techniques looks promising but pose major challenges, such as achieving high resolution and integrating multiple materials evenly in multi-layer configurations. For effective fabrication, the right materials selection and materials' compatibility with the 3D printing process are vital aspects. Recent developments in 3D printing techniques have led to promising outcomes with the hope of opening up future explorations on improving the capabilities of 3D printing or integrating multiple printing systems for the commercial production of optrode arrays.

Once a fabrication protocol is established, whether utilizing conventional cleanroom processes or additive manufacturing, process development and re-design will be required to scale up fabrication to pilot manufacturing lines and commercial operations. Consideration should be given to which steps need to be automated, substituted, and/or retained, keeping in mind that a reduction in the number of manufacturing work instructions is advantageous. The cost-effectiveness of manual steps vs the automation of steps also needs to be analyzed. Finally, the translation to regulated products should also include the costs of setting up quality control systems and adequate manufacturing practice.

### G. Biocompatibility

For any new medical device to enter the market, comprehensive biological evaluation plans (BEPs) are required by regulatory bodies across the globe. The International Standards Organization (ISO) 10993–1 outlines the need for a structured BEP that identifies risks and proposes appropriate approaches to managing these risks via biocompatibility testing.^349^

When evaluating any device, the contact duration and nature and type of tissue contact needs to be taken into account. Permanent, implantable devices are the highest risk category and typically require more extensive testing across the range of possible endpoints. Flexible optrode arrays have potential applications ranging from short-term surface contacting devices through to permanent implantable devices. This review focuses on optrodes for electrophysiology, which have potential contact durations from acute through to chronic and a broad range of tissue types and nature of contact from surface optrodes to tissue penetrating forms. Thus, the following discussion will center on the highest risk categories of tissue contact.

**FIG. 7.**
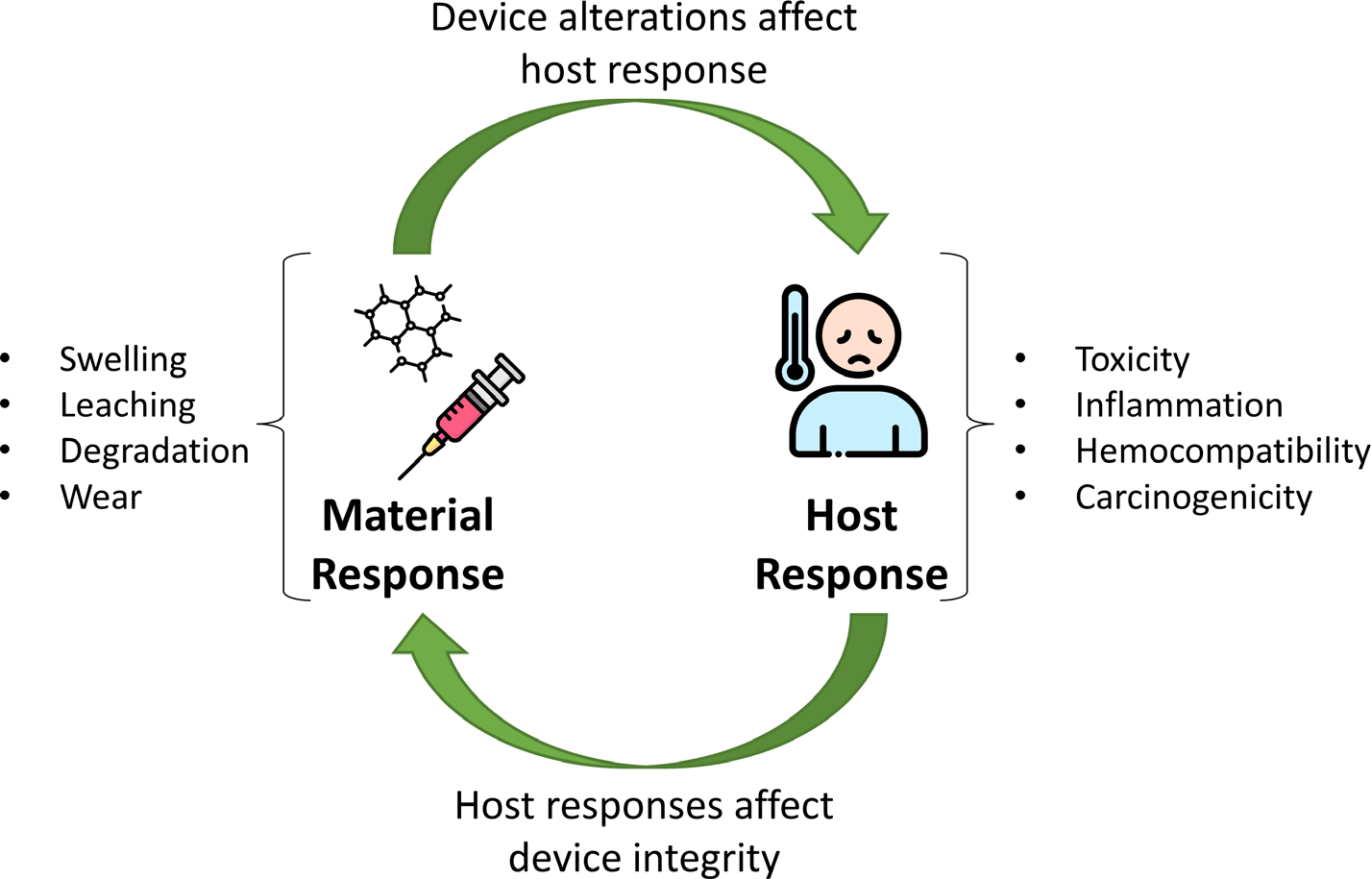
Critical considerations in evaluating safety risks of medical devices include the material and host responses and interactions between these aspects. Examples of material responses that may occur are shown as are the major categories of host responses; these examples are not exhaustive.

Biological performance considerations when developing any new medical device encompass the material responses to the host environment, the adverse host, or device recipient responses to the device, and the interplay between these two aspects (as shown in Fig. 7). Each of these elements needs to be considered when developing a plan for biocompatibility testing. It is important to note that this is not a box ticking exercise, rather it is a careful evaluation of the likely biocompatibility risks, given the device components and the devices' operating environment is in the body.

Material responses, mechanical degradation, biostability, fluid and/or lipid absorption and effects of sterilization all need to be considered. A key risk in a flexible optrode array relates to the mechanical degradation due to cyclic stress the device is likely to experience. Given that most tissue contacting materials proposed in a flexible array are polymers manufactured as thin films, this repeated stress can potentially cause fatigue of materials and release of components that are intended to be hermetically sealed within the device.^350^ These include materials that have potential biocompatibility risks such as liquid crystals, organic conductors, and nanoparticulate metals, carbon, and polymers.

Polymers such as PDMS, commonly referred to as silicone, is often applied as an insulator in bioelectronic medical devices. While silicone has excellent biological performance, it has a relatively low toughness and can experience deterioration of properties after implantation, particularly when manufactured as thin films.^351^ Alternative polymers proposed for flexible optrodes include parylene-C and polyimide, which can be manufactured as ultrathin, tough insulators. However, there remain concerns with fabrication and the potential for delamination and leakage that may limit chronic implantation.^315,352^

Biostability is another risk that must be addressed for any new polymeric component intended for longer-term implantation. The combined effects of mechanical and biological stresses on an implanted polymer can lead to catastrophic stress cracking as observed in poly(urethanes) used to fabricate pacemaker leads.^353,354^ The biological factors relevant to biostability encompass the cellular and humoral host responses that are outlined in more detail below. Such long-term degradation processes are not well understood for many of the polymers proposed for dielectric components, and their safe use may be restricted to acute applications until definitive evidence of stability is gathered.

Fluid uptake is another key risk consideration when analyzing potential material responses. This is not a significant issue for metal, ceramic and carbon components; however, all polymers have some degree of fluid uptake.^355^ Fluids, including lipids found in the blood, can be absorbed into polymers and may have a plasticizing effect or may facilitate extraction of small molecules from the polymer. Both of these phenomena can cause alterations in materials properties that can cause device failure. Catastrophic failure occurred in early heart valves when the silicone formulations used experienced significant lipid uptake resulting in valve occlusion.^356^ The lesson learned from this was that polymer formulations require careful design, and appropriate testing needs to be conducted (including in the targeted tissue) to measure fluid uptake and the resulting material responses.

Many applications use accelerated *in vitro* testing regimes to demonstrate material responses including fluid uptake, leaching of extractable components, and durability. Examples of this can be found in accelerated testing of heart valves that experience similar cyclic stresses to cardiac electrophysiology optrodes, albeit for significantly longer time periods.

Finally, another concern with respect to material responses is the potential for material property changes following sterilization. Polymers, in particular, are susceptible to polymer chain degradation or increased cross-linking following gamma irradiation and sterilization using ethylene oxide.^357^ These can impact on polymer stiffness via embrittlement, degradation, and mass loss due to chain scission and can impact on fluid uptake *in vivo*, which can result in swelling and dimensional change of the device.

Where optrodes may differ from electrodes is in the materials interfacing tissues. In most bioelectronic applications, the electrodes are typically fabricated using platinum or platinum alloys. Deterioration in these metal electrodes can occur, particularly where electrical stimulation and high levels of charge injection are required. This deterioration is caused by a combination of factors such as high charge densities and various biological factors.^358^ Given the nature of the tissue interface in optrodes, the risk of such deterioration may be reduced particularly in optrode arrays for delivery of light for optogenetic, direct light, and nanoparticle-mediated thermal modulation, as these techniques are purely light-based and eliminate the need for charge delivery.

Although biocompatibility testing is not directly concerned with device efficacy, this may be adversely affected by alteration of the material or device properties following implantation. An example of this is in the increased resistance observed in flexible spinal cord stimulators as a result of gold track microcracking due to applied strain.^359^

Considering host responses, both the geometry and the chemistry of the device can cause inflammation, toxic responses, and adverse blood responses. Device geometry is largely controlled in the design stage, and ensuring that the shape, size, and topography cause minimal tissue irritation or trauma is a key factor in minimizing host responses. Efforts to develop more flexible arrays largely address concerns relating to mechanical mismatch between device and tissue. As already noted, material responses may cause alterations in the device geometry. Swelling can change the device size and shape, and degradation may cause significant alteration in topography at both micro and macro levels. Finally, material breakdown or failure of the device hermeticity can result in release of particles spanning nano to macro scales.

The most likely host responses to these geometrical factors in non-blood contacting applications are chronic inflammatory reactions resulting from tissue trauma, or the presence of particulates and, to a lesser extent, carcinogenicity. Put simply, inflammation is a physiological response to injury and tissue damage, and many of the biocompatibility testing categories measure such inflammatory responses. These include implantation testing with biological endpoints of acute and chronic inflammation and fibrosis, as well as irritation and intracutaneous reactivity, which encompasses measurement of edema and erythema, indicators of an acute inflammatory response. Inflammatory responses can also contribute to biological effects measured using other biocompatibility tests including systemic toxicity and subacute, subchronic, and chronic toxicity, although there are other biological effects that can contribute to these including immune-mediated reactions and direct toxicity resulting in cell and tissue necrosis.

The risk of carcinogenicity always needs to be analyzed where materials with no history of toxicological testing or use *in vivo* are proposed. Asbestosis and silicosis illustrate the severe potential health and safety impacts of small particles with specific chemistries.^360^ Given the long time course and high cost of carcinogenicity testing, the alternatives for introducing new materials that may release particles into the body are by conducting genotoxicity testing and providing extensive analyses of the physical and chemical risk factors. Given the challenges of such approaches, most devices are over-engineered so that any potentially toxic or carcinogenic physical and chemical components are isolated through hermetic sealing in containers that are rigorously tested for long-term stability.

Chemical risk factors need to be evaluated for all device materials. Materials are typically selected based on prior evidence of their chemical safety in identical application types. However, flexible optrodes are likely to comprise a range of materials that do not have a history of use in medical devices; therefore, extensive risk analysis will be required. The chemistry can also be altered *in vivo* as already mentioned, and this can result in degradation byproducts, particulates, or leachables being released systemically. Where there is uncertainty in risk levels and exclusion of tests cannot be justified, materials will need to be extensively tested through a comprehensive biological performance regime. This regime is somewhat similar to that described for geometrical factors, encompassing tests evaluating inflammatory reactions and primary and immune-mediated toxicity and carcinogenicity. Blood contacting devices are not considered here given the nature of tissue contact for reviewed electrophysiology recording and stimulating devices being mainly on the heart's epicardium and neural tissue rather than in direct or indirect blood contact. If the device is designed to directly interface with blood, then the level of complexity increases significantly due to hemocompatibility risks and the potential for critical systemic impacts.

In summary, the uncertainty relating to short- and long-term toxicity of optrode components and to the capacity of flexible encapsulation systems to remain stable *in vivo* presents key challenges when innovating in the field of flexible electronics and optrode-based devices.

## VII. FUTURE DIRECTIONS

This review has detailed many novel and emerging optically related technologies for recording and stimulation. A particular emphasis was placed on design approaches for soft, flexible, and conformable constructs. These include liquid crystal electro-optical transducers, plasmonic probes, as well as genetic and non-genetic photostimulation. Despite great potential, the true impact of these technologies is yet to be realized. They parallel the limitations of MEAs with respect to the need for creating high-density, high-channel-count bidirectional sensing and stimulation arrays. However, the prospects of MEAs exhibiting a disruptive advance in this space is limited due to the size constraints imposed by the necessity of electrical wiring to address each individual electrode in an MEA.

Groups have tried to bypass the interconnection constraints by directly interfacing embedded electronics, which also helps address other issues with MEAs, including electrode impedance scaling inversely with electrode size, and lower SNRs due to high electrode impedances. However, the use of active electronics brings about other significant constraints. Such electronics need to be powered and encapsulated to allow for their safe operation in close apposition to biological tissue. These constructs are also inherently rigid and inflexible, making it infeasible to be made into a soft or conformable structure. In contrast, the optical technologies described herein, while currently in more nascent forms, nevertheless have huge potential to revolutionize electrophysiological recordings. Assuming they can be arrayed into conformable constructs, they will herald the next generation of brain–computer interfaces as described below.

Photonic concepts are still relatively foreign in the fields of electrophysiology and biomaterials but are standard in telecommunications systems worldwide. Such optical fiber communication systems operate at 1550 nm. Hence, closer integration of these fields will revolutionize the optical electrophysiological systems, conferring the benefits of decades of advancements in the telecommunications field on biopotential recordings and stimulation of excitable tissue. This is especially the case for NIR stimulation and liquid crystal electro-optical transducers, which utilize light wavelengths in the 1400–1700 nm range. Similarly, well-established techniques in optical telecommunication engineering, such as wavelength division multiplexing and Bragg gratings, can achieve an order of magnitude greater channel density on optrode arrays compared to state-of-the-art MEAs. We foresee a future whereby flexible optical-electrical arrays and well-established telecommunication techniques merge to enable unprecedented high-channel, high-resolution arrays for electrophysiology.

In the field of optical electrophysiology, one of the key areas of focus for future research is the development of novel flexible materials for use in biomedical devices and associated fabrication methods for optical electrodes. We have detailed some examples of these materials and the attendant considerations around biocompatibility. This includes exploring how new materials can confer flexibility and conformability, so, for example, they could be designed to conform to the complex geometries of the heart, brain, or other neural tissues. In the future, such approaches would be applied to devices such as liquid crystal electro-optical transducers, plasmonic probes, and genetically encoded sensors for improved detection and manipulation of neural activity. The design of such materials requires intense effort, due to the trade-off we described between optical performance and flexibility, and the critical need to ensure that the materials or their encapsulants can be long-term implanted without the risk of chronic inflammation or tissue damage. It is for this reason that by far the majority of the technologies described herein are in preclinical testing stages. Two exceptions, which have moved to human trials, are photobiomodulation using NIR light and photovoltaic retinal prosthesis to restore patterned vision in the profoundly vision impaired.

At the moment, flexible endoscopic or portable microscopic systems for optogenetic manipulation and other light-based techniques for modulating or recording the electrical activity of cells *in vivo* are scarce.^335–337^ Assuming that appropriate flexible optrode array constructs are developed, future trends will see them being applied in ever expanding biological and clinical application areas. This could include their incorporation into flexible endoscopy systems for imaging of cell and tissue structures for histology assessment, and for real-time identification of tissue types. Such systems would have application in the identification of cancerous tissue, for example, glioblastoma removal during brain surgery. Another application area for such minimally invasive endoscopic devices would be in real-time cardiac mapping and ablation for treatment of cardiac arrhythmias.

Beyond the development of novel materials and flexible arrays, there is a need to improve the signal quality of optrodes for improved sensitivity and signal to noise ratio (SNR). This will be important for achieving recordings that are on par with electrical systems. One promising though quite new technology in this area is the use of liquid crystal electro-optical transducers, which can provide recordings with actual magnitude measurements rather than relative measures.

Looking further into the future, there is potential for the development of nanoscale optical electrodes and photonic devices for precise manipulation and recording of excitable activity at the cellular and subcellular level. This would enable unprecedented spatial and temporal resolution and could lead to significant advancements in our understanding of neural and cardiac function and the development of new therapies for neurological and cardiac rhythm disorders.

## VIII. CONCLUSIONS

In summary, the future of optical electrodes and high-density arrays for electrophysiology and brain–machine interfaces is promising. Continued research and development in novel materials, fabrication methods, and flexible array design will likely lead to significant improvements in signal quality, spatial resolution, and recording density, paving the way for new insights into excitable tissue function and for the development of more effective diagnostics and therapeutic tools for clinical disorders of electrophysiological origin.

## Data Availability

Data sharing is not applicable to this article as no new data were created or analyzed in this study.
